# Rhizobial migration toward roots mediated by FadL-ExoFQP modulation of extracellular long-chain AHLs

**DOI:** 10.1038/s41396-023-01357-5

**Published:** 2023-01-10

**Authors:** Yuan-Yuan Ji, Biliang Zhang, Pan Zhang, Liu-Chi Chen, You-Wei Si, Xi-Yao Wan, Can Li, Ren-He Wang, Yu Tian, Ziding Zhang, Chang-Fu Tian

**Affiliations:** 1 State Key Laboratory of Agrobiotechnology, and College of Biological Sciences, China Agricultural University, Beijing, China; 2 MOA Key Laboratory of Soil Microbiology, and Rhizobium Research Center, China Agricultural University, Beijing, China; 3 Shenzhen Institute of Synthetic Biology (iSynBio) Shenzhen Institute of Advanced Technology (SIAT), Chinese Academy of Sciences, 518055 Shenzhen, China

**Keywords:** Microbial ecology, Functional genomics, Bacterial genetics

## Abstract

Migration from rhizosphere to rhizoplane is a key selecting process in root microbiome assembly, but not fully understood. *Rhizobiales* members are overrepresented in the core root microbiome of terrestrial plants, and here we report a genome-wide transposon-sequencing of rhizoplane fitness genes of beneficial *Sinorhizobium fredii* on wild soybean, cultivated soybean, rice, and maize. There were few genes involved in broad-host-range rhizoplane colonization. The *fadL* mutant lacking a fatty acid transporter exhibited high colonization rates, while mutations in *exoFQP* (encoding membrane proteins directing exopolysaccharide polymerization and secretion), but not those in *exo* genes essential for exopolysaccharide biosynthesis, led to severely impaired colonization rates. This variation was not explainable by their rhizosphere and rhizoplane survivability, and associated biofilm and exopolysaccharide production, but consistent with their migration ability toward rhizoplane, and associated surface motility and the mixture of quorum-sensing AHLs (N-acylated-L-homoserine lactones). Genetics and physiology evidences suggested that FadL mediated long-chain AHL uptake while ExoF mediated the secretion of short-chain AHLs which negatively affected long-chain AHL biosynthesis. The *fadL* and *exoF* mutants had elevated and depleted extracellular long-chain AHLs, respectively. A synthetic mixture of long-chain AHLs mimicking that of the *fadL* mutant can improve rhizobial surface motility. When this AHL mixture was spotted into rhizosphere, the migration toward roots and rhizoplane colonization of *S. fredii* were enhanced in a diffusible way. This work adds novel parts managing extracellular AHLs, which modulate bacterial migration toward rhizoplane. The FadL-ExoFQP system is conserved in *Alphaproteobacteria* and may shape the “home life” of diverse keystone rhizobacteria.

## Introduction

Despite an ever-enlarging catalog of prokaryote species [1], few taxa are globally abundant in soils [2]. Soil characteristics play a more important role than plant genotypes in determining the composition of root-associated bacterial communities [3, 4]. In this context, many studies have further identified a subset of community members which are reproducibly associated with a plant species across multiple geographic sites of different conditions, such as rice [5], citrus [6], sugarcane [7], and diverse legume species [8]. Available evidences support the establishment of a multi-step model for root microbiome assembly, sequentially from bulk soil to rhizosphere to rhizoplane to endosphere, in which rhizoplane is a selecting gate [5, 9]. This multi-step pattern has been described extensively, but the underlying gene-environment interactions, shaping the distribution and abundance of rhizobacteria in different niches (bulk soil, rhizosphere, and rhizoplane), remain largely unexplored [10–12]. This knowledge gap impedes the ultimate evolutionary explanation for the “home life” of rhizobacteria in these ecological niches [12].

Genome-wide identification of rhizoplane fitness genes of model rhizobacteria has been greatly speeded up by the Tn-seq assay under controlled conditions [13–18]. However, functional characterizations of these fitness genes at different colonization steps were not addressed in these high-throughput studies. The current multi-step colonization model involves chemotaxis toward roots, attachment and subsequent formation of multi-cellular biofilm on rhizoplane, which is supported by cumulative reverse genetics evidences from various rhizobacteria [19]. Chemical gradients of root exudates are supposed to serve as crucial conditions (e.g., signalling function) and resources (nutritional value), which differentially interact with motile bacteria to shape the migration process toward the root niche [20, 21]. Ligands directly recognized by bacterial chemoreceptors are mostly unknown [22], though chemoeffector function has been proposed for organic acids, carbohydrates, sugar alcohols, amino acids, and plant hormones [23]. Upon rhizoplane attachment mediated by diverse bacterial adhesins [24], motile bacteria gradually transit into sessile forms, which is followed by the growth and maturation of matrix-embedded bacterial communities (exopolysaccharides (EPSs), eDNA, eRNA, proteins, lipids, and other biomolecules), i.e., the multi-cellular biofilm [25]. Biofilm is the “home” for multiple cells of the same or different species, which communicate with each other via highly-conserved quorum-sensing signals N-acyl homoserine lactones (AHLs) [26]. By contrast, it remains elusive to what extent the density-dependent quorum-sensing process is involved in migration toward the root niche [19, 27, 28]. This is essential for understanding and engineering root microbiome assembly [29, 30].

*Rhizobiales* and *Burkholderiales* belong to the core root microbiome of terrestrial plants [31, 32]. These orders are enriched with both beneficial rhizobia associated with legumes and diverse plant pathogens [8], which have been intensively studied for their molecular interactions, post rhizoplane colonization, with hosts [33–36]. Rhizobia are characterized by their intracellular infection and nitrogen fixation in legume nodule cells [33], and can be considered as a pioneer of legume root microbiota. This distinct inter-kingdom symbiosis is initiated by specific sensing of legume flavonoids by rhizobial transcriptional regulator NodD and subsequent activation of other *nod* genes involved in the biosynthesis of lipochitooligosaccharides (known as Nod factors), which are in turn specifically recognized by host receptor to induce downstream infection and nodule morphogenesis [36]. Flavonoids were also proposed as a chemoeffector for rhizobia based on earlier studies [20], which has recently been questioned with new evidence from cosolvents usually used to dissolve flavonoids [37]. Moreover, rhizobia are recurrently reported as endophytes of diverse plant species, and their plant growth promotion effects on cereals such as rice [38, 39], wheat [40, 41], and maize [42] have been demonstrated. Although molecular interactions between rhizobia and their hosts post rhizoplane colonization have been intensively studied [34, 36], it remains largely unknown how beneficial rhizobia successfully colonize diverse terrestrial plants.

To investigate rhizobial machineries involved in broad-host-range rhizoplane colonization, we focused on *Sinorhizobium fredii* that is a broad-host-range facultative microsymbiont of diverse legume species [43, 44]. Tn-seq was used for genome-wide analysis of rhizoplane colonization genes of *S. fredii* CCBAU25509 (hereafter SF2) on wild soybean (*Glycine soja* W05), cultivated soybean (*Glycine max* cv. JD17), maize (*Zea mays* cv. ZD958), and rice (*Oryza sativa* cv. Nipponbare). This allowed identification of a robust list of broad-host-range rhizoplane colonization genes. Within this list, reverse genetics verified that mutations in *fadL* (encoding an outer membrane transporter) and *exoFQP* (encoding membrane proteins directing polymerization and secretion of EPSs) led to improved and impaired broad-host-range rhizoplane colonization ability, respectively. Characteristics associated with rhizosphere and rhizoplane survivability (EPS and biofilm production) and migration toward roots (swimming and surface motility) were determined for related mutants. Finally, we revealed that the opposite rhizoplane colonization competence between *fadL* and *exoFQP* mutants was consistent with their contrasting migration ability toward roots, and associated surface motility and extracellular quorum-sensing long-chain AHLs. AHL quantification also allowed us to propose a working model for migration toward roots involving FadL-ExoFQP-dependent modulation of extracellular long-chain AHL homeostasis. This model was further verified in a rhizoplane colonization experiment using a synthetic mixture of long-chain AHLs.

## Materials and methods

### Bacterial strains and growth conditions

Bacterial strains and plasmids used in this study are listed in Table S1, and primers are shown in Table S2. Rhizobia were grown at 28 °C in TY medium [45]. *Escherichia coli* strains were grown at 37 °C in Luria-Bertani (LB) medium. *Agrobacterium tumefaciens* was grown at 28 °C in LB medium. Concentrations of antibiotics were described earlier [45, 46]. The Bioscreen C (Oy Growth Curves Ab Ltd, Raisio, Finland) was used to determine growth curves of test strains.

### Mutant library construction

To construct saturated transposon insertion mutant libraries, the mariner transposon-borne pSAM_Sf derived from pSAM_Bt [47] was introduced into SF2 via conjugation. After growth, the mating spot was scraped and resuspended in 0.85% NaCl solution. This mating mix (100 µL per plate) was further spread on 600 TY agar plates (90 × 90 mm) containing nalidixic acid (30 μg/ml), trimethoprim (10 μg/ml) and kanamycin (50 μg/ml) using sterile glass beads and incubated for 3 days at 28 °C. Around 600,000 colonies were harvested generating the input transposon insertion mutant library for subsequent experiments. Three independent libraries were constructed and directly used in three independent experiments.

### Collection of rhizoplane and control samples

**Fig. 1:**
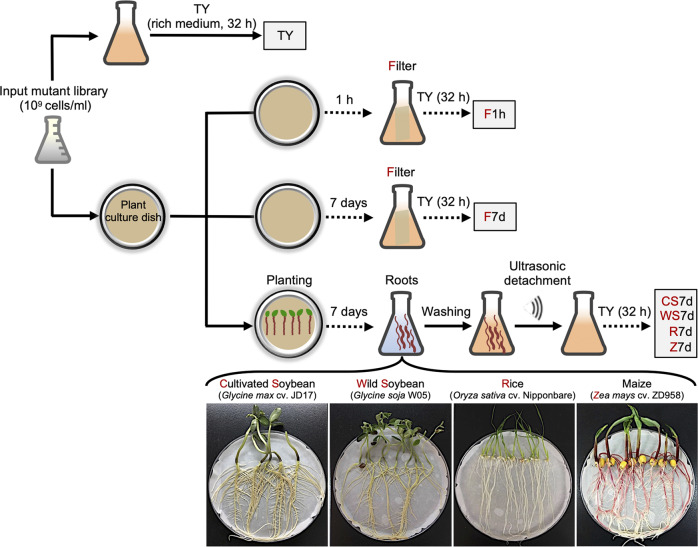
Schematic overview for collecting output libraires for the Tn-seq analysis of root colonization arsenal of *Sinorhizobium fredii* on legume and cereal plants. Three independent *mariner* transposon insertion libraries of *S. fredii* CCBAU25509 (SF2) were used as input inoculants in three independent experiments. Their 1h-post-inoculation (F1h) and 7d-post-inoculation (F7d) samples on the filter of plant culture dish were used as control samples to those rhizoplane samples of four test plant species (CS7d, WS7d, R7d, and Z7d). To facilitate Tn-seq library construction, all output samples were subject to cultivation in the TY rich medium for 32 h, and the input libraries were cultivated under the same condition as control (TY).

Seeds of wild soybean, cultivated soybean, rice, and maize were treated with 95% ethanol for 30 s, and surface-sterilized in 17% NaClO (wt/vol) solution for 3 min (with wild soybean seeds pretreated by concentrated sulfuric acid for 3 min), then washed five to seven times using autoclaved deionized water and germinated on 0.8% agar plates at 28 °C in the dark for 48 h. To minimize false negatives in fitness gene screen, the input library was resuspended in 0.85% saline solution at OD_600_ = 1. The input library was inoculated onto the filter paper of plant culture dish (0.8% agar; low-N nutrient solution medium [45]). At the same time, 2-day-old seedlings were transferred to these culture dishes. Roots were then harvested at 7 dpi (days post inoculation), pooled, weighed, washed 5 times with 0.85% NaCl solution, and per 10 g were suspended in 15 ml 0.85% NaCl solution. After ultrasound treatment (50 Hz, 30 s for twice), the suspension was incubated in 200 ml TY medium with antibiotics for 32 h to facilitate further Tn-seq library construction for these rhizoplane samples (WS7d, CS7d, R7d, and Z7d). Control samples included 32 h TY cultures of input library (TY) and those TY cultures of filter papers collected from the plant culture dish at 1 hpi (hour post inoculation; F1h) or 7 dpi (F7d). Three independent mariner transposon insertion libraries were used as input libraries in three independent experiments (Fig. 1).

### Tn-seq library construction

DNA from the collected samples was extracted using a TIANamp Bacteria DNA kit (Tiangen) and subjected to enzymatic fragmentation with MmeI (New England Biolabs). About 3 μg of qualified DNA was digested with 3 μl (6 units) MmeI in a total volume of 200 µL for 2.5 h at 37 °C. Then 2 μl Calf Intestinal Alkaline Phosphatase (CIP; New England Biolabs) was added and the reaction system was incubated for 1 h at 37 °C. The double-strand adapters with different barcodes were ligated to the restriction fragments purified using a QIAquick PCR purification kit (Qiagen), and incubated overnight at 16 °C. Transposon insertion sites flanking sequences were enriched by PCR using Universal and P7-Tn primers, and Q5 DNA polymerase (New England Biolabs) (2 min at 98 °C; 22 cycles at 98 °C for 10 s, 72 °C for 25 s, and 72 °C for 30 s; followed by a final extension at 72 °C for 5 min). PCR products were run on a 1.8% agarose gel and purified using a QIAquick Gel Extraction Kit (Qiagen). DNA was quantified with a Nanodrop and subject to single-end sequencing on the NextSeq 550AR platform (ANORAD Gene Technology Co., Ltd).

### Tn-seq data analysis

Raw reads were filtered using fqgrep (https://github.com/indraniel/fqgrep) to identify the adapter and transposon, and genomic DNA adjacent transposon was extracted. Bowtie was used to map reads to SF2 genome [48], generating a *.sam* output file which was then used to produce a .*wig* format file of the aligned genomic DNA using summarize_mappings.py (https://github.com/elijweiss/Tn-seq). The resulting *.wig* files were analyzed by the “Resampling” method of TRANSIT with the TTR normalization method (trimmed total reads) [49]. Only insertions within the 5–95% ORFs were considered to minimize the potential effect of non-disrupting insertions [50]. Either individual input libraries or F1h samples were used as controls.

### Reverse genetics procedure

To verify Tn-seq results, representative genes were mutated by insertion of pCM351 [51] derivatives carrying an internal fragment of individual target genes with primers listed in Table S2. All pCM351 derivatives were verified by Sanger sequencing, and then conjugated into SF2. For in-frame deletion of *fadL*, *exoF*, *traI*, *sinI*, and *flaA* in SF2 or various backgrounds, a seamless assembly cloning kit (Taihe Biotechnology, Beijing, China) was used to construct pJQ200SK [52] derivatives as described previously [53], with corresponding primers listed in Table S2. The correct engineered plasmids harbored by positive clones were verified using PCR and Sanger sequencing, and then conjugated into rhizobia. Single-crossover clones resistant to gentamicin were further subject to counter selection for double recombinants using 5% sucrose. To construct complementary mutant strains, the fragments containing corresponding coding sequences and upstream promoter regions were cloned into pBBR1MCS-2 [54]. The pBBR1MCS-2 derivatives with correct cloned sequences were introduced to rhizobia by conjugation. To generate GFP- and mCherry-tagged *S. fredii* strains, pRJPaph-bjGFP and pRJPaph-mChe [55] were delivered to *S. fredii* by conjugation. All insertion mutants, in-frame deletion mutants, complementary mutant strains, and tagged strains were verified by colony PCR and Sanger sequencing.

### Competitive nodulation and rhizoplane colonization

To determine nodule occupancy of rhizobia, the mutants were mixed with their parent strains at 1:1 (OD_600_ = 0.2) and inoculated on host plants grown in vermiculite moistened with the low-N nutrient solution medium (alfalfa seeds were sterilized using the same method as wild soybean seeds). At 30 dpi, nodules were surface sterilized (95% ethanol for 30 s, and 17% NaClO for 3 min) and nodule isolates were identified by their growth on TY plates with corresponding antibiotics, and PCR with primers targeting strain-specific fragments.

For the rhizoplane colonization experiment, the same system described for collecting Tn-seq samples was used with a reduced inoculation density as described below. The overnight rhizobial cultures were adjusted to an OD_600_ of 0.2 in 0.85% saline solution which was used as an inoculant. At 7 dpi, colony forming units (CFUs) in rhizoplane samples or on the filter paper were determined by incubating diluted samples on TY agar plates with corresponding antibiotics. To determine rhizoplane survivability of rhizobia on wild soybean roots, seedlings with root length of 3 cm were placed in 300 µl rhizobial solution of OD_600_ = 0.2 for 10 s, then air-dried and transferred to the plant culture dish. Roots were then harvested at 1 hpi and 7 dpi, and CFUs in rhizoplanes samples were determined as described above. To characterize the migration process from rhizosphere to rhizoplane, 2 µl wild-type or mutants of OD_600_ = 0.8 (around 1.6 × 10^6^ CFUs) were inoculated symmetrically at 1 cm, 2 cm, 3 cm, and 4 cm away from wild soybean seedlings in the plant culture dish. At 7 dpi, CFUs in filter paper samples at inoculation sites and rhizoplane samples were determined as described above. This symmetric inoculation procedure was also used to test the diffusible effect of long-chain AHLs (2 µl solution containing 45 ng 3-OXO-C12-HSL, 52 ng C14-HSL, and 1 μg 3-OXO-C14-HSL) on rhizoplane colonization of SF2 or mutants (inoculant: around 1.6 × 10^6^ CFUs), i.e., with the inoculation positions of both AHLs and bacteria varying at symmetric distances to wild soybean seedlings.

### Biofilm formation assay

The biofilm formation was determined as described previously [56] with modifications. Overnight TY cultures of rhizobia were centrifuged, washed twice using 0.85% NaCl solution, and adjusted to OD_600_ = 0.1 in TY. The resultant culture of 100 µl was inoculated into 96-well polystyrene microtiter plates (8 wells per strain) which were then sealed with parafilm and set for 48 h in a growth chamber at 28 °C. Bacterial growth was determined at OD_600_ on a microplate reader. Planktonic bacteria were removed with pipette, and the plates were washed 3 times with 0.85% NaCl solution. The wells were emptied and stained with 150 µl of 0.1% (w/v) crystal violet for 10 min and rinsed three times with distilled water. Microtiter plate was inverted on tissues to remove any excess liquid and air-dried. The remaining crystal violet was solubilized by adding 150 µl of 95% (v/v) ethanol and incubating for 15 min. Finally, 125 μl of the crystal violet/ethanol solution from each well was transferred into a 96-well plate, and OD_590_ was measured with a microplate reader.

### Quantification of exopolysaccharides

Exopolysaccharides (EPSs) were purified and quantified as previously described [57]. Briefly, bacteria were cultured in the M9 medium for 5 days and harvested, and OD_600_ was measured for standardization. The supernatant containing EPSs was obtained by centrifugation (2449 g for 30 min), and the high molecular weight (HMW) fraction was precipitated by adding 3:1 volume of 95% cold ethanol. The HMW precipitate was collected by centrifugation (2449 *g* for 30 min) and seven volumes more of ethanol was added to the obtained supernatant to precipitate the low molecular weight fraction. These two fractions then were lyophilized and weighed.

### Swimming and surface motility assays

To assess the swimming of rhizobia, bacterial colonies were transferred with a sterile toothpick into the center of swimming agar plates (TY, 0.3% agar with Congo red). For surface motility, 2 µl of overnight culture (OD_600_ = 0.8) of test strains were spot-inoculated centrally on the surface motility agar plate (TY, 0.5% agar with Congo red). The plates were incubated face up at 28 °C for 7 days, and the maximum diameters of a corresponding swimming or surface motility colony were determined.

To test the competitive surface motility of mutants and SF2, mCherry-labeled mutants and GFP-labeled SF2 were mixed at different ratios (10%, 30%, 50%, 70%, and 90%) and incubated on surface motility plates (TY, 0.5% agar) for 1 week. To test the competitive swimming motility of the mCherry-labeled *flaA* mutant and GFP-labeled SF2, two strains were mixed at 1:1 ratio and inoculated into the center of swimming agar plate (TY, 0.3% agar). Seven days later, each colony was observed with Leica fluorescence stereo microscope (the excitation light source: 488 nm for detection of GFP-labeled SF2 and 546 nm for detection of mCherry-labeled mutants).

To analyze the effect of long-chain AHLs on the surface motility of bacteria, each mCherry-labelled strain was mixed at 1:1 ratio with its corresponding unlabeled strain to a concentration of OD_600_ = 0.8, and then spot-inoculated with long-chain AHLs (2 µl bacterial solution contains 45 ng 3-OXO-C12-HSL, 52 ng C14-HSL, and 1 μg 3-OXO-C14-HSL) on the center of the surface motility agar plate (TY, 0.5% agar with Congo red). Seven days later, the colony diameter was recorded, and pictures were taken with Leica fluorescence stereo microscope (excitation light source: 546 nm).

### Qualitative and quantitative detection of AHLs by an indicator strain

*Agrobacterium tumefaciens* KYC55(pJZ372)(pJZ384)(pJZ410) [46] was used as an indicator strain to detect extracellular AHLs from *S. fredii* strains. Test strains were cultured on surface motility plates with or without x-gal for 3 days, and then KYC55(pJZ372)(pJZ384)(pJZ410) (OD_600_ = 0.1) was looped outside rhizobial colony. At 4 dpi of KYC55(pJZ372)(pJZ384)(pJZ410), the plates containing x-gal were photographed, and the plates without x-gal were used for quantification of β-galactosidase activity of KYC55(pJZ372)(pJZ384)(pJZ410) to reflect the extracellular AHL content of rhizobia.

### HPLC and mass spectrometry analysis of intra- and extracellular AHLs

To measure intra- and extracellular short- and long-chain AHLs, rhizobia were incubated in the TY medium at 28 °C for 32 h (stationary phase). The supernatant containing extracellular AHLs was obtained by centrifugation (2449 *g* for 30 min). The pellet was washed twice with 0.85% NaCl, resuspended, sonicated, and then centrifuged to obtain the supernatant containing intracellular AHLs. The resulting samples were extracted twice with chromatographic grade ethyl acetate in equal volumes, and the organic phase was evaporated. The extracts were dissolved in 1 mL methanol:water (1:1 v/v) containing 0.1% (v/v) formic acid, and microfiltered (0.22 µm). The resulting sample of 1 µL was injected onto the HPLC system equipped with a TACQUITY UPLC HSS T3 column (2.1 × 100 mm, 1.8 µm particle size) (Thermo Scientific, Waltham, MA. USA). Eluents were formic acid 0.1% in water (solvent A) and in acetonitrile (solvent B). Flow rate was set at 300 μL/min, the mixture percentage changed from 40% solvent B to 98% solvent B during the first 9 min, maintained for 2 min, and then the column returned to the starting conditions. All of the AHLs molecules have a high serine lactone ring, which forms a characteristic fragment with m/z of 102 in HPLC mass spectrometry [58]. According to this principle, the HPLC-Q-Exactive-PRM method was established. Analytical standards (purity > 99%) of C8-HSL, 3-OXO-C8-HSL, C12-HSL, 3-OXO-C12-HSL, C14-HSL, 3-OXO-C14-HSL were purchased from Sigma (Darmstadt, Germany). The 1 µg/ml solutions were serially diluted to 1/4, 1/16, 1/256, and 1/1024. Mass spectrometric analyses were performed on a Q Exactive HF-X quadrupole trap mass spectrometry (Thermo Scientific, Waltham, MA. USA). Precursor ion-scanning experiments were performed in positive-ion mode to monitor for m/z 102.

## Results and discussion

### Identification of broad-host-range rhizoplane colonization genes by Tn-seq

This work was focused on SF2 harboring a typical multipartite genome of *Sinorhizobium* (chromosome, chromid, and symbiosis plasmid) [59]. To perform genome-wide survey of rhizoplane colonization genes of SF2 (Fig. 1), the input mutant library was inoculated on filter paper of plant culture dish, and output mutant libraries were collected from filter papers at 1 h post inoculation (F1h) and 7 days post inoculation (dpi; F7d), and from rhizoplane of cultivated soybean (CS7d), wild soybean (WS7d), rice (R7d), and maize (Z7d) at 7 dpi. To facilitate Tn-seq library construction, all output mutant libraries were subject to 32 h cultivation in the TY rich medium, with input libraries cultivated at the same condition as control (TY). Tn-seq revealed that transposon insertion density in three input and 21 output samples ranged from 57.03 to 86.99% (Table S3), which are above the threshold of 50% insertion density for a good Tn-seq dataset [49]. A reproducible rhizosphere effect was observed in three independent experiments (Fig. S1), i.e., rhizoplane samples (CS7d, WS7d, R7d, and Z7d) consistently formed distinct clusters compared to those of TY, F1h, and F7d. A considerable signature of three independent input libraries was also identified (Data S1, Data S2, and Fig. S1). These results highlight that stochastic variations among multiple independent input libraries should be considered before making conclusions on gene fitness, which has been largely overlooked in earlier studies based on just one input library [49].

Based on gene fitness scores of rhizoplane samples (CS7d, WS7d, R7d and Z7d) compared to corresponding F1h datasets (Fig. S2A; Data S2), 93, 91, 127, and 206 genes were identified as rhizoplane colonization genes for test plants of cultivated soybean, wild soybean, maize, and rice, respectively, accounting for 1.4–3.1% of the SF2 genome (*p* values < 0.01). This range is similar to those reported for rhizoplane colonization genes in *Sinorhizobium meliloti* (2%) [60], *Rhizobium leguminosarum* bv. *viciae* (2.9%) [17], *Agrobacterium tumefaciens* (2.9–4%), [18], *Pseudomonas aeruginosa* (1.6%) [15], *Pseudomonas simiae* (2%) [13], *Pseudomonas* sp. WCS365 (3.8%) [14], *Azoarcus olearius* (2.2%), and *Herbaspirillum seropedica*e (2.7%) [16]. Among these rhizoplane fitness genes, 8, 8, 21, and 82 genes were specific to cultivated soybean, wild soybean, maize, and rice, respectively (Fig. S2A and Data S2.1). Moreover, 43 genes were reproducibly identified as a rhizoplane colonization gene in three independent experiments for at least one test plant species (Fig. S2B, and Data S2.2), which were therefore considered as a robust list of rhizoplane colonization genes for further investigation. The control samples from filter papers (F7d) formed a distinct cluster compared to those rhizoplane samples in the hierarchical clustering analysis (Fig. S2B). Since a notable noise was observed for *c09590* in R7d.3 and Z7d.3 datasets, a *c09590* mutant was constructed and co-inoculated with SF2 at 1:1 ratio under the same condition used for Tn-seq sample collection. Moreover, a mutant for *c04390* was also constructed and used as a positive control in the same experiment. The *c09590* and *c04390* mutants exhibited significantly impaired broad-host-range rhizoplane colonization ability compared to SF2 (*p* values < 0.001; Fig. S3), supporting the Tn-seq results (Fig. S2B).

There were 41 out of 43 genes making positive contribution to rhizoplane colonization. These genes are involved in repair of DNA (UvrA) and protein (Pcm), translation (YchF and LepA), respiration (seven proteins related to cytochrome C), phosphonate metabolism (PhnP), antioxidant activity (SodA), uptake of ferric iron (c03990 and c04080) and oligopeptide (b57060, b57070, and b57080), lipoprotein attachment (c19900), secretion of EPS (ExoF, ExoP, and ExoQ), anabolism for tryptophan (TrpA, TrpB, TrpD, and TrpE), glutamate (GltA, GltB, and ArgD), leucine (LeuA and LeuB), methionine (MetA), IMP (PurC and PurF), AMP (PurA) and dUMP (Dcd). Within this gene list, homologs of 22 genes were also identified as rhizoplane colonization players (Data S2.2) in *S. meliloti*-alfalfa [60], *P. simiae*-*Arabidopsis thaliana* [13], *Pseudomonas* sp. WCS365-*A. thaliana* [14], *P. aeruginosa*-maize [15], *A. olearius*/*H. seropedicae*-*Setaria viridis* [16], *R. leguminosarum*-pea [17], or *Agrobacterium tumefaciens*-tomato pairs [18]. In contrast, genes encoding a putative anti-sigma factor (RsbU) and the only known outer membrane hydrophobics transporter FadL [61–64] negatively regulated rhizoplane colonization of SF2, with *fadL* having a stronger effect (Fig. S2B). In a recent Tn-seq survey of colonization genes of *Aquitalea magnusonii* on the floating whole plant of duckweed, the *fadL* mutants were enriched in the output pool indicating a negative impact on colonization [65].

**Fig. 2:**
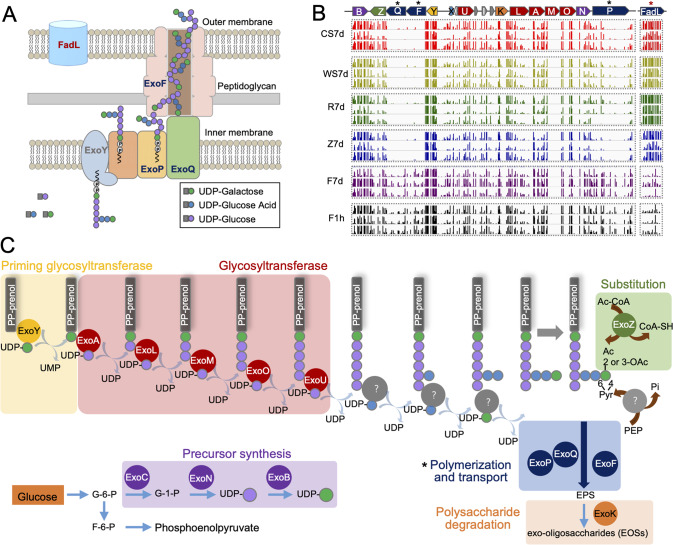
Exopolysaccharide polymerization and transport machinery rather than biosynthesis arsenals are required for rhizoplane colonization. **A** Predicted subcellular localization of ExoP, ExoQ, ExoF, and FadL. **B** The *exo* gene cluster directing exopolysaccharide biosynthesis, transport, and degradation in SF2, and its transposon insertion frequency in three independent experiments (rows) under different conditions. The results of *fadL* is also shown. **C** The predicted pathway of exopolysaccharide biosynthesis, transport, and degradation in SF2. G-6-P glucose-6-phosphate, F-6-P fructose-6-phosphate, Ac acetyl group, Ac-CoA Acetyl-CoA, PEP phosphoenolpyruvate.

These 43 rhizoplane colonization genes have a biased replicon distribution in SF2 multipartite genome: 81.4% on chromosome, 18.6% on chromid, and 0% on symbiosis plasmid. This is consistent with earlier metabolic modeling evidence from *S. meliloti* showing that *Sinorhizobium* chromosome and chromid contribute to rhizosphere fitness [66], while the symbiosis plasmid encoding genes mainly involved in establishing and maintaining nitrogen-fixing symbiosis with legume plants [59]. Nevertheless, symbiotic optimization by chromosomal and chromid genes has been reported [34, 67, 68], among which EPS biosynthesis genes on chromid have been intensively studied regarding their role in optimizing both infection process of *Sinorhizobium* on legume plants [69, 70] and biofilm formation in vitro [71]. In this work, among 8 chromid genes involved in rhizoplane colonization (Fig. S2B), *exoF*, *exoP*, and *exoQ* encode membrane proteins directing EPS polymerization and secretion (Fig. 2A) [72–77]. It was intuitively unexpected that various EPS biosynthesis genes within the same gene cluster of *exoF*, *exoP*, and *exoQ* were not essential for rhizoplane colonization (Fig. 2B, C). This implies a working model that other unknown physiological function of ExoF, ExoP, and ExoQ play a more important role than EPS production in rhizoplane colonization.

### Opposite contribution to rhizoplane colonization by FadL and ExoFQP

**Fig. 3:**
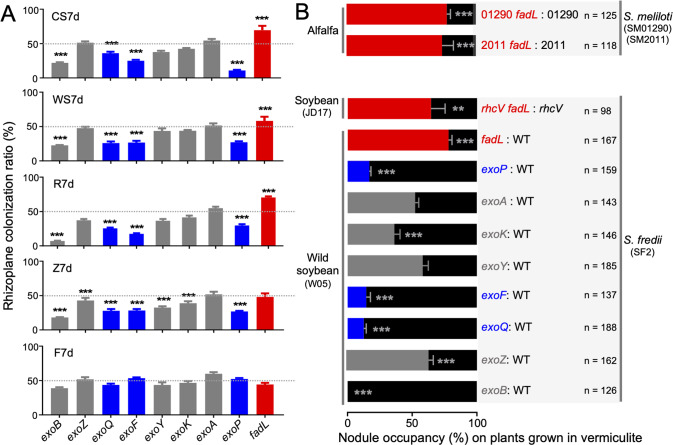
Rhizoplane colonization and nodule occupancy capability of the *exoF*, *exoQ*, *exoP*, and *fadL* mutants. **A** Impaired rhizoplane colonization ability of the *exoF*, *exoQ*, and *exoP* mutants, and increased performance of the *fadL* mutant. Test mutants were individually co-inoculated with the wild-type strain SF2 at 1:1 ratio under the same conditions used for Tn-seq (Fig. 1; 1 ml inoculant of OD_600_ = 0.2 herein). **B** Impaired nodule occupancy, on legume plants grown in vermiculite, by the *exoF*, *exoQ*, *exoP* mutants, and increased performance of the *fadL* mutants. The wild-type SF2 (WT) forms effective nodules on *Glycine soja* (wild soybean W05) but not on *Glycine max* cv. JD17 (cultivated soybean), while its *rhcV* mutant can establish effective nodules on JD17. The *fadL* mutants of *S. meliloti* strains SM01290 (CCBAU01290; BioSample: SAMN02388829) and SM2011 (BioSample: SAMN02603522) were also tested on their host *Medicago sativa* (alfalfa). Significant difference in competitive rhizoplane colonization and nodule occupancy between individual mutants and WT is indicated (one sample *t*-test; theoretical mean = 0.5; **, *p* < 0.01; ***, *p* < 0.001). Error bars represent SD of three biological replicates (nodules from more than 15 plants were analyzed, and the number of test nodules is shown).

As described above, among broad-host-range rhizoplane fitness genes (Data S2.2 and Fig. S2), mutations of *fadL* led to outstanding high rhizoplane colonization rates, while mutations in *exoFQP* involved in EPS polymerization and secretion, but not those in *exo* genes required for EPS biosynthesis, led to severely impaired colonization rates. To verify the opposite role of FadL and ExoFQP in rhizoplane colonization, mutants of *fadL*, *exoF*, *exoP*, and *exoQ* were constructed. Moreover, mutants of five *exo* genes involved in different steps of EPS biosynthesis and degradation were constructed for comparison: *exoB* (precursor synthesis), *exoY* (priming glycosyltransferase), *exoA* (glycosyltransferase), *exoZ* (substitution), and *exoK* (polysaccharide degradation) (Fig. 2C). These mutants were individually co-inoculated with SF2 at 1:1 ratio under the same condition for Tn-seq (Fig. 1). Their competitive ability in rhizoplane colonization was generally consistent with the Tn-seq results. The *fadL* mutant was more competitive than SF2 in the CS7d, WS7d, and R7d treatments (Fig. 3A; *p* values < 0.001) but indistinguishable from SF2 in Z7d. By contrast, the *exoF*, *exoP*, and *exoQ* mutants were outcompeted by SF2 on four plant species (Fig. 3A; *p* values < 0.001).

**Fig. 4:**
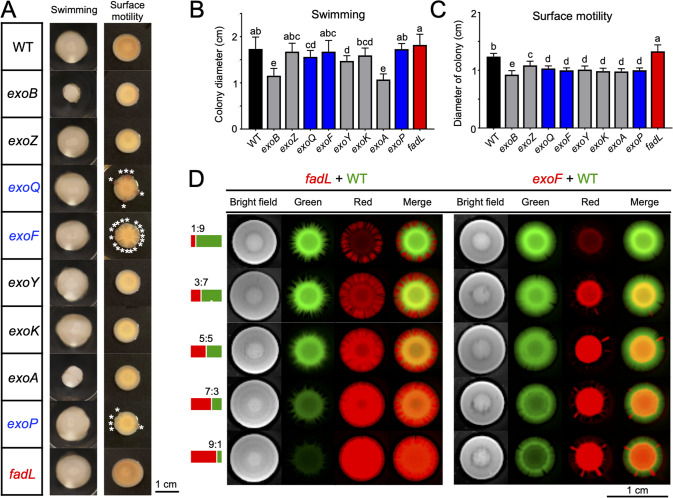
Rhizoplane colonization ability in line with variations in surface motility rather than swimming ability. **A** Swimming (left, 0.3% agar) and surface motility (right, 0.5% agar) abilities on the TY plate with Congo red. *, notable cracks in colonies of the *exoQ*, *exoF*, and *exoP* mutants. **B**, **C** Diameter of colonies under swimming (**B**) and surface motility (**C**) conditions as shown in (**A**). **D** Fluorescence stereo microscopy pictures of surface motility ability of the *fadL* mutant (red) and the *exoF* mutant (red) compared to the wild-type SF2 (WT; green) in an inoculant mixture (mixed in different ratios as follows: 1:9, 3:7, 5:5, 7:3, and 9:1). **B**, **C** Different letters indicate significant difference between means (±SEM; ANOVA followed by Duncan’s test, alpha = 0.05; three independent experiments).

The *exoY*, *exoA*, *exoZ*, and *exoK* mutants were indistinguishable from SF2 in the CS7d, WS7d, and R7d treatments, while the *exoY*, *exoZ*, and *exoK* mutants showed an impaired competition ability in Z7d (33–43%; *p* values < 0.001), though to a lesser extent than the *exoF*, *exoP*, and *exoQ* mutants (27–28%). The notable exception was the *exoB* mutant which was outcompeted by SF2 herein (Fig. 3A) while no depletion of *exoB* mutants was observed in Tn-seq data from rhizoplane samples (Fig. 2B). ExoB, encoding a UDP-glucose 4-epimerase, synthesizes UDP-galactose [78] which might be provided by other mutants within the mixture of mutant library. This hypothesis is supported by several lines of evidence: (1) a cryptic *uxe* gene in *Sinorhizobium* encodes a bifunctional UDP-sugar 4-epimerase, catalyzing UDP-xylose/UDP-arabinose and UDP-glucose/UDP-galactose interconversions, and its overexpression can functionally replace *exoB* [79]; (2) this *uxe* gene is however generally repressed by the global silencer MucR in wild-type *Sinorhizobium* strains [79–81]; (3) a considerable number of transposon insertions were observed in two *mucR* copies (c08920 and a45250; 377-758 insertions per gene) within Tn-seq data of rhizoplane samples (Data S1). The drastic rhizoplane colonization defect of the *exoB* mutant compared to the *exoY*, *exoA*, *exoZ*, and *exoK* mutants (Fig. 4A) may be due to the fact that UDP-galactose, synthesized by ExoB, is a precursor for multiple galactose-containing polysaccharides (lipopolysaccharides, succinoglycan and galactoglucan) [82].

### Opposite contribution to competitive nodulation by FadL and ExoFQP

Since competitive nodulation between commercial rhizobial inoculants and indigenous rhizobia is a long-lasting issue in agriculture practices [83], these mutants were further tested for their nodule occupancy ability on plants grown in vermiculite. The wild-type SF2 can form effective nodules on many wild soybean accessions and some soybean landraces but not on certain modern soybean cultivars [67, 84]. When these mutants were inoculated on wild soybean plants, only the *exoB* mutant showed significant defects in nodulation and symbiotic performance regarding shoot dry weight (Table S4). Moreover, the *exoB* mutant was totally outcompeted by SF2 in competitive nodulation experiment (1:1 inoculation ratio; Fig. 3B). The *exoF*, *exoP*, and *exoQ* mutants also showed a significant defect in nodule occupancy (13–17%; Fig. 3B), while only *exoK*, among the other *exo* mutants, showed an impaired nodule occupancy to a lesser extent (36%). The *fadL* (78%) and *exoZ* (63%) mutants occupied more nodules than SF2. As the *exoY*, *exoA*, *exoZ*, and *exoK* mutants were all indistinguishable from SF2 regarding rhizoplane colonization on wild soybean plants (Fig. 3A), the role of ExoZ and ExoK in competitive nodulation may be not due to their predicted functions in EPS modification and degradation [73, 85].

We have previously reported that mutations in *rhcV*, encoding an essential component of type three secretion system in SF2, allow an improved nodulation and symbiotic performance on certain commercial soybean cultivars such as JD17 [84, 86]. When *fadL* was further mutated in the *rhcV* mutant background, the *rchV fadL* double mutant exhibited a higher nodule occupancy (61%) than the *rhcV* mutant on JD17 (Fig. 3B; *p* < 0.01). We further tested the effects of *fadL* mutation in a model strain SM2011 [87] and an inoculant strain SM01290 [88] of *S. meliloti* associated with alfalfa. These two *fadL* mutants occupied more nodules than their wild-type strains SM2011 and SM01290 (Fig. 3B; 74–78%; *p* values < 0.001). Collectively, contrasting nodule occupancy ability between the *fadL* mutant and the *exoF*, *exoP*, and *exoQ* mutants were in line with their opposite rhizoplane colonization ability.

### Variation in surface motility of the *fadL*, *exoF*, *exoP*, and *exoQ* mutants

In order to uncover potential traits associated with the contrasting rhizoplane colonization ability of test mutants (Fig. 2 and Fig. 3A), their growth curves (Fig. S4A), EPS production (Fig. S4B), biofilm formation (Fig. S4C), swimming and surface motility (Fig. 4A–C) were compared. The *exo* and *fadL* mutants were not significantly different from each other on their growth curves in the TY rich medium (Fig. S4A). All *exo* mutants except *exoZ* produced less EPS (both high- and low- molecular weight compositions; Fig. S4B), which were in line with their biofilm formation ability (Fig. S4C). These results support the view that EPS is important matrix components of biofilm [25]. Notably, the *exoF, exoP*, and *exoQ* mutants were not distinguishable from the other *exo* mutants except *exoZ*, and the *fadL* mutant had similar EPS production and biofilm formation ability as SF2 (Fig. S4B, C). Apparently, these characteristics of growth curves, EPS and biofilm production could not explain the observed variation in rhizoplane colonization competence of the *fadL* and *exo* mutants (Fig. 2 and Fig. 3A). This is likely due to the fact that biofilm is the “home” for multiple cells [25], among which some family members (such as certain *exo* mutants) can effectively co-colonize rhizoplane with other EPS producers in the root microbiota.

Given the important role of motility in migration toward roots, swimming and surface motility of test mutants were compared (Fig. 4A–C). The *fadL* mutant had a higher surface motility ability than SF2 (Fig. 4C; ANOVA followed by Duncan’s test, alpha = 0.05). All *exo* mutants exhibited a lower surface motility regarding the diameter of colonies on the semisolid plate (0.5% agar with Congo red; Fig. 4C) while the *exoF*, *exoP*, and *exoQ* mutants had notable radial cracks in their colonies (marked by * in Fig. 4A), which were not observed for the other *exo* mutants (Fig. 4A). The variation in the swimming ability of test mutants were however not consistent with their rhizoplane colonization ability (Fig. 4B). To further verify the contrasting surface motility phenotypes, the GFP-labeled SF2 and mCherry-labeled *fadL* or *exoF* mutants were mixed at different ratios 1:9, 3:7, 5:5: 7:3 and 9:1 (Fig. 4D), and the higher and lower surface motility ability of the *fadL* and *exoF* mutant, respectively, was demonstrated across all inoculation ratios. The surface motility ability of the *fadL*, *exoF*, *exoP*, and *exoQ* mutants can be fully restored in their corresponding complementary mutant strains (Fig. S5).

Available literature suggests that surface motility on the test semisolid plate (0.5% agar) can be flagellum-dependent swarming and/or flagellum-independent motility processes e.g., pilus-dependent twitching, adhesin-dependent gliding, and sliding driven by the pressure of growing cells and facilitated by additional factors (e.g., surfactant, EPS, and surface protein) in a species- and resource-dependent manner [89, 90]. In this work, no known genes on flagellum, pilus or adhesin were found in the list of broad-host rhizoplane fitness genes (Fig. S2 and Data S2.2). For example, when four *flaA* genes encoding flagellar filament subunits were deleted (Fig. S6A), the resultant *flaA* mutant showed an impaired flagellum-dependent swimming ability as expected (TY plates with 0.3% agar; Fig. S6B–D; *p* < 0.01), but was indistinguishable with SF2 regarding surface motility (TY plates with 0.5% agar; Fig. S6B, D) and in the rhizoplane colonization ability assay (Fig. S7). In short, the variation in surface motility, likely sliding and/or other unknown movement mechanisms, was in line with the opposite rhizoplane colonization ability between the *fadL* mutant and the *exoF*, *exoP*, and *exoQ* mutants.

### Variation in migration ability toward roots

**Fig. 5:**
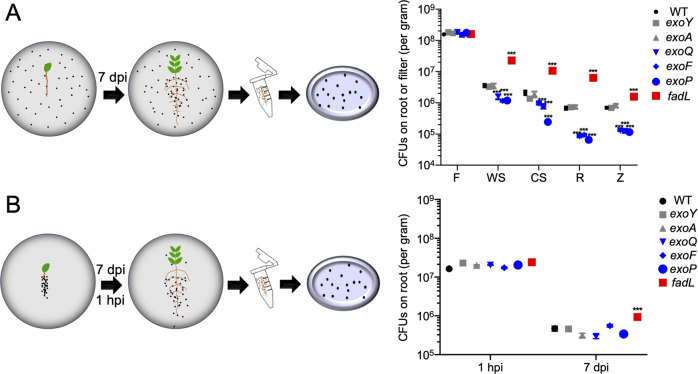
Contrasting rhizoplane colonization abilities not due to rhizoplane survivability. **A** Rhizoplane colonization ability of individual strains. F filter without plants, WS wild soybean, CS cultivated soybean, R rice, Z maize. **B** Rhizoplane survivability of individual strains on WS roots at 7 dpi (days post inoculation). Samples at 1 hpi (hours post inoculation) were used for comparison. Significant difference compared to the wild-type strain SF2 (WT) is indicated (*t-*test; ***, *p* < 0.001). Error bars represent SD of three biological replicates (not visible for those small SD values).

The evidences mentioned above (Figs. 2–4) suggest a correlation between surface motility ability and competitive rhizoplane colonization. Here we further evaluated the rhizoplane colonization ability of individual mutants of *fadL*, *exoF*, *exoP*, and *exoQ*, with the *exoY*, *exoA*, and SF2 strains as controls. The *fadL* mutant showed a significantly higher rhizoplane colonization ability on wild soybean, cultivated soybean, rice, and maize, while the *exoF*, *exoP*, and *exoQ* mutants, but not the *exoA* and *exoY* mutants, showed significant impairment in rhizoplane colonization (Fig. 5A; *p* values < 0.001). By contrast, these mutants showed similar survivability on the filter paper of culture dish in the absence of plants (Fig. 5A; *p* > 0.05). The number of colony-forming units (CFUs) on rhizoplane showed a global host-dependent pattern (Fig. 5A), consistent with the variation in root exudates of different plants species [20] and their effects on root microbiome assembly [21]. Nevertheless, the opposite rhizoplane colonization ability between the *fadL* mutant and the *exoF*, *exoP*, and *exoQ* mutants was not host-dependent. Therefore, the following experiments on rhizosphere effects were only performed on one plant species (wild soybean).

The observed variation in rhizoplane colonization rates (Fig. 2, Fig. 3, and Fig. 5A) can involve survivability in rhizosphere and on rhizoplane, and migration from rhizosphere to rhizoplane, which however has not been well addressed in published Tn-seq studies of rhizoplane colonization genes [13–18]. The test *exo* and *fadL* mutants didn’t show growth defect in the TY rich medium (Fig. S4A) and survivability defect on filter paper of plant culture dishes (Fig. 5A), but it remained unknow if this would hold true on rhizoplane. To answer this question, test strains were individually inoculated on seedling roots before planting in culture dishes (Fig. 5B). The number of CFUs on rhizoplane at 7 dpi was significantly higher for the *fadL* mutant compared to the other test strains (SF2, *exoY*, *exoA*, *exoQ*, *exoF*, and *exoP*; *p* values < 0.001), while the *exo* mutants and SF2 were not significantly different from each other (Fig. 5B). These results suggest that rhizoplane survivability at least partially accounts for the superior rhizoplane colonization ability of the *fadL* mutant, but does not correlate with colonization defects of the *exoF*, *exoP*, and *exoQ* mutants.

**Fig. 6:**
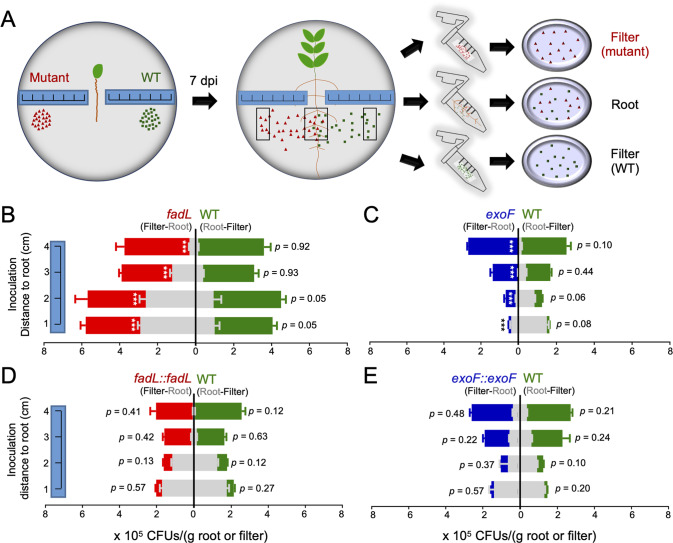
Genotype-dependent variation in migration ability toward roots. **A** A schematic overview showing rhizosphere survivability assay of the wild-type SF2 (WT; green) and its derivatives (red), which were inoculated in a symmetrical way at 1 cm, 2 cm, 3 cm, and 4 cm away from the roots. **B–E** Colony forming units (CFUs) from the inoculation sites (filter) and rhizoplane (root) were tested at 7 dpi (days post inoculation). Significant difference compared to WT is indicated (*t*-test; ***, *p* < 0.001). Error bars represent SD of three biological replicates.

These results further raised a question whether both survivability and surface motility would be the rhizosphere processes underlying the contrasting rhizoplane colonization abilities among the *fadL*, *exoF*, *exoP*, and *exoQ* mutants. To answer this question, the *fadL* or *exoF* mutant, and SF2 was individually spotted at 1/2/3/4 cm away from seedlings in a symmetrical way (Fig. 6A). CFUs at the inoculation spot and on rhizoplane were determined at 7 dpi. For all strains, the farther the inoculation site was away from seedlings, the lower the number of CFUs on rhizoplane (Fig. 6B–E; grey bars), which indicated a distance-decay pattern for rhizoplane colonization rates. This provides direct evidence for a spatial pattern of bacteria-root interaction efficiency, which is taken for granted in various models of rhizosphere effects but rarely tested [19, 20, 91]. Across 1–4 cm sites, these two mutants showed no significant difference in survivability compared to SF2 (Fig. 6B, C; *p* values > 0.05). Regardless of survivability across these sites, the *fadL* and *exoF* mutants had higher (153–223% more CFUs than SF2; *p* values < 0.001; grey bars on the left panel of Fig. 6B) and lower (75–98% lesser CFUs than SF2; *p* values < 0.001; grey bars on the left panel of Fig. 6C) rhizoplane colonization rates, respectively, compared to SF2 (grey bars on the right panels of Fig. 6B, C). All rhizosphere phenotypes associated with these two mutants can be restored in their corresponding complementary mutant strains (Fig. 6D, E). These results are consistent with their contrasting surface motility ability (Fig. 4 and Fig. S5). Collectively, the variation in rhizoplane colonization ability among the *fadL* and *exoF* mutants, and SF2 is mainly modulated by their surface motility in the migration process from rhizosphere to rhizoplane.

### Extracellular quorum-sensing long-chain AHLs enhance migration toward roots

**Fig. 7:**
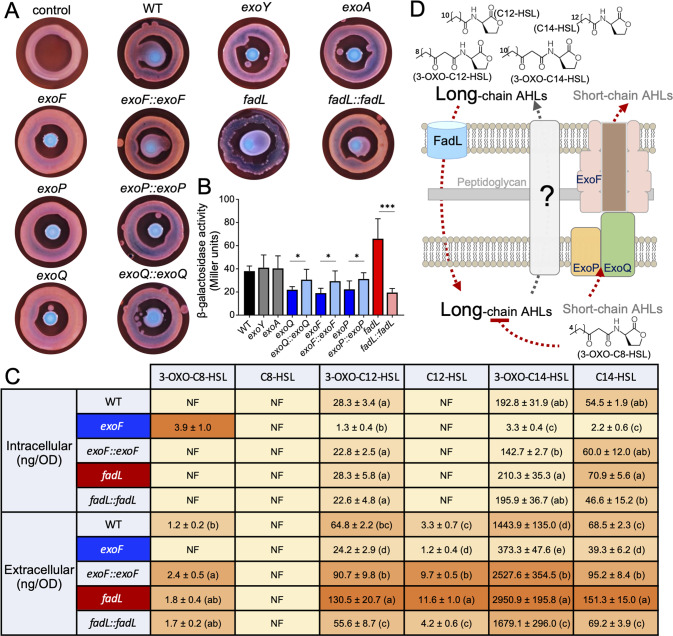
Genotype-dependent variation in extracellular and intracellular AHLs. **A** Detection of extracellular AHLs from rhizobia (central colony) using biosensor *A. tumefaciens* KYC55(pJZ372)(pJZ384)(pJZ410) (circle) on the TY plate (0.5% agar, Congo red, and X-gal). **B** β-galactosidase activity of *A. tumefaciens* KYC55(pJZ372)(pJZ384)(pJZ410) cells collected from (**A**). **C** Intra- and extracellular AHL concentrations determined using the HPLC-Q-Exactive-PRM-MS approach. NF, not found. **D** A working model for the transport of long- and short-chain AHLs through FadL and ExoFPQ integrated in membranes. A predicted repression of long-chain AHL biosynthesis by short-chain AHLs is shown. Significant difference between means is indicated in (**B**) (*, *p* < 0.05; ***, *p* < 0.001; *t*-test), and (**C**) (different letters in brackets; ANOVA followed by Duncan’s test, alpha = 0.05) based on three independent experiments. Error bars represent SEM (**B**) or SD (**C**).

The available evidence suggests that the homolog of long-chain fatty acid transporter FadL in *S. meliloti* may promote long-chain AHL (with acyl chains containing at least 12 carbons) uptake [92], and long-chain AHLs can function as biosurfactants facilitating surface motility of *Rhizobium etli* on the semisolid plate (yeast extract mannitol medium with 0.75% agar) [93]. However, direct measurement of AHL mixture from the *fadL* mutant was not available yet. We therefore examined the extracellular content of AHLs in SF2, mutants of *fadL* and *exo*, and their complementary mutant strains by using an AHL indicator strain *A. tumefaciens* KYC55(pJZ372)(pJZ384)(pJZ410) [46] (Fig. 7A). This indicator strain, lacking the AHL biosynthesis ability, overexpresses the AHL receptor TraR which can activate the expression of P*traI*-*lacZ* fusion upon interaction with AHL molecules of various chain lengths, saturation levels, and oxidation states [46]. On the surface motility plate, the *fadL* mutant made *A. tumefaciens* KYC55(pJZ372)(pJZ384)(pJZ410) expressing more β-galactosidase than SF2 (Fig. 7B) and thus cleaving more X-gal into the blue product (Fig. 7A). By contrast, the *exoQ*, *exoF*, and *exoP* mutants had a reduced level of extracellular AHLs and the *exoY* and *exoA* mutants were similar to SF2 (Fig. 7A, B). The increased and reduced levels of extracellular AHLs from the *fadL* and *exoF/exoP/exoQ* mutants can be restored in their complementary mutant strains under test conditions (Fig. 7A, B). To our knowledge [72–77, 94], this may represent the first report of the involvement of EPS secretion system in modulating extracellular AHLs.

To get further insight into the mixture of AHLs associated with the *fadL* and *exoF* mutants, intra- and extracellular AHLs were determined using the HPLC-Q-Exactive-PRM-MS approach (Fig. 7C). A small amount of a short-chain AHL 3-OXO-C8-HSL was detected inside the *exoF* mutant, but undetectable in cells of the other test strains. By contrast, no extracellular 3-OXO-C8-HSL was detected for the *exoF* mutant, but detectable for the other strains, among which the complementary mutant strain of the *exoF* mutant had more extracellular 3-OXO-C8-HSL than SF2 (ANOVA followed by Duncan’s test, alpha = 0.05). The *exoF* mutant was also characterized by its lower intracellular (3-OXO-C12-HSL, 3-OXO-C14-HSL, and C14-HSL) and extracellular long-chain AHLs (3-OXO-C12-HSL, C12-HSL, 3-OXO-C14-HSL, and C14-HSL; ANOVA followed by Duncan’s test, alpha = 0.05) than SF2. Its complementary mutant strain had higher extracellular long-chain AHLs than SF2 (C12-HSL, 3-OXO-C14-HSL, and C14-HSL; ANOVA followed by Duncan’s test, alpha = 0.05), while being indistinguishable from SF2 in their intracellular long-chain AHLs. Collectively, these results suggest a model where ExoF is essential for secretion of the short-chain AHL 3-OXO-C8-HSL, intracellular accumulation of which in the *exoF* mutant can result in down-regulation of the biosynthesis and extracellular level of long-chain AHLs (Fig. 7D). Earlier efforts show that a multidrug efflux system MexAB-OprM from *P. aeruginosa* is involved in active secretion of long-chain AHLs, and that the BpeAB-OprB efflux system from *Burkholderia pseudomallei* is required for secretion of AHLs of various length [95, 96]. The short-chain AHLs in other bacteria have been hypothesized to diffuse freely across membranes [97]. Apparently this long-last hypothesis on passive diffusion of AHLs, particularly for those short-chain AHLs, deserves more extensive investigations.

**Fig. 8:**
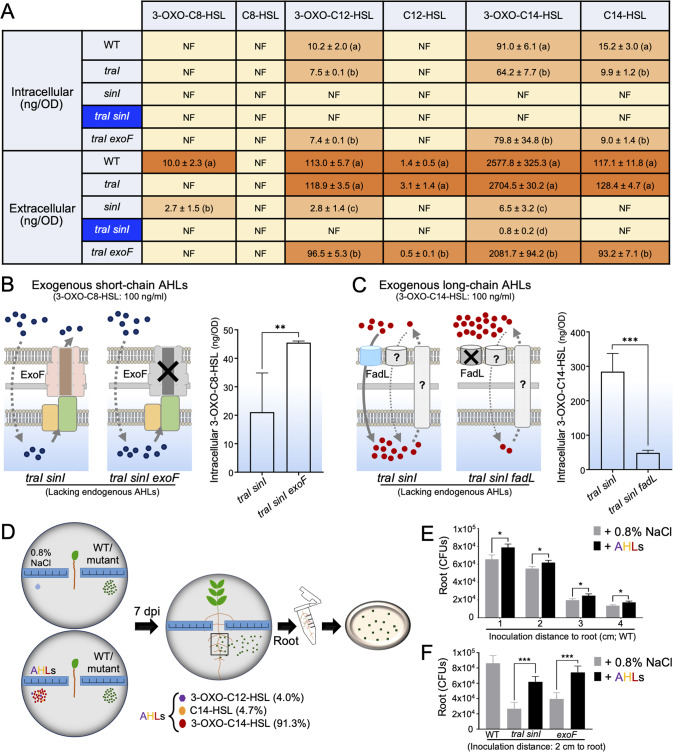
Exogenous long-chain AHLs enhance rhizobial migration ability toward roots. **A** Intra- and extracellular AHL concentrations determined using the HPLC-Q-Exactive-PRM-MS approach. NF not found. Different letters indicate significant differences between means (±SD, three independent experiments) based on ANOVA followed by Duncan’s test, alpha = 0.05. **B** Intracellular short-chain AHLs of *traI sinI* and *traI sinI exoF* mutants in the presence of 100 ng/ml exogenous 3-OXO-C8-HSL. **C** Intracellular long-chain AHLs of *traI sinI* and *traI sinI fadL* mutants in the presence of 100 ng/ml exogenous 3-OXO-C14-HSL. **D–F** Exogenous long-chain AHLs enhance rhizobial migration ability toward roots (2 μl long-chain AHL solution, containing 45 ng 3-OXO-C12-HSL, 52 ng C14-HSL, and 1 μg 3-OXO-C14-HSL, according to the extracellular AHL composition detected from the *fadL* mutant in Fig. 7C). **D** A schematic overview of experimental design showing inoculation of rhizobia (WT/mutants) and AHLs (or 0.8% NaCl as the negative control) at symmetric distances to root. **E**, **F** Long-chain AHLs enhance rhizoplane colonization by WT (**E**), the *traI sinI* and *exoF* mutants (**F**). Significant difference between means (±SEM, three independent experiments) are indicated in (**B–F**) (*, *p* < 0.05; **, *p* < 0.01; ***, *p* < 0.001; *t*-test).

In *S. fredii*, the *traI* and *sinI* genes are essential for the biosynthesis of short- and long-chain AHLs, respectively [98]. Indeed, no 3-OXO-C8-HSL was detected from the *traI* and *traI exoF* mutants of SF2 (Fig. 8A). The depleted levels of intracellular (1.7–4.6% of SF2) and extracellular (25.9–57.4% of SF2) long-chain AHLs of the *exoF* mutant (Fig. 7C) were considerably restored by further deletion of *traI* (Fig. 8A; 58.9–87.7% and 79.6–85.4% for intracellular and extracellular long-chain AHLs, respectively). Moreover, the *traI sinI* double mutant, unable to produce endogenous AHLs (Fig. 8A), accumulated lesser intracellular 3-OXO-C8-HSL than the *traI sinI exoF* triple mutant in the presence of 100 ng/ml exogenous 3-OXO-C8-HSL (Fig. 8B). Collectively, these results support the important role of ExoF in secreting short-chain AHLs, and that intracellular accumulation of short-chain AHLs negatively modulates the biosynthesis of long-chain AHLs (Fig. 7D).

In contrast, the *fadL* mutant had a similar intracellular level of AHLs compared to SF2, while characterized by the highest extracellular long-chain AHLs (Fig. 7C; ANOVA followed by Duncan’s test, alpha = 0.05). The intra- and extracellular levels of short-chain AHL 3-OXO-C8-HSL were similar between the *fadL* mutant and SF2. The extracellular level of long-chain AHLs of the *fadL* mutant can be restored in its complementary mutant strain (Fig. 7C). Moreover, the *traI sinI fadL* triple mutant showed a significant defect in 3-OXO-C14-HSL uptake compared to the *traI sinI* double mutant in the presence of 100 ng/ml exogenous 3-OXO-C14-HSL (Fig. 8C). These findings provide more insightful evidences to support the hypothesis of FadL as an uptake system for long-chain AHLs [92] (Fig. 7D).

Apparently, the contrasting rhizoplane colonization ability and surface motility among the *fadL* and *exoF* mutants, and SF2 are in concord with their variations in the mixture of extracellular AHLs. It has been demonstrated that long-chain AHLs can function as biosurfactants promoting surface motility of *R. etli* [93]. We wondered if a synthetic mixture mimicking the extracellular composition of AHLs from the *fadL* mutant would improve surface motility and rhizoplane colonization rates of SF2 and its various mutants. Thus 3-OXO-C12-HSL (4.0%), 3-OXO-C14-HSL (91.3%), and C14-HSL 3 (4.7%) were synthesized and mixed as a mixture of AHLs. On the semisolid TY plate (0.5% agar), this mixture of AHLs significantly improved surface motility of SF2, *flaA*, *exoF*, and *traI sinI* mutants (Fig. S8; *p* values < 0.001). The *exoF*, *traI sinI*, and *sinI* mutants (*p* values < 0.001), but not the *flaA* and *traI* mutants, showed impaired root colonization rates in the symmetrical inoculation experiments (Fig. S7 and Fig. 6). When the synthesized mixture of long-chain AHLs was symmetrically inoculated as SF2, *traI sinI*, and *exoF* at the same distance to roots (Fig. 8E, F), rhizoplane colonization rates of these strains were significantly enhanced compared to the 0.8% NaCl control (*p* values < 0.05). This diffusible effect of long-chain AHLs was detectable at 1 cm, 2 cm, 3 cm or 4 cm away from roots (Fig. 8E, F). Moreover, this AHL mixture can also significantly improve the survivability of SF2 at 1 cm and 2 cm sites (Fig. S9; *p* values < 0.001). Similarly, the *fadL* mutant can also enhance the survivability of SF2 when these two strains were symmetrically inoculated on either side of seedlings (Fig. 6B), though the *fadL* mutant, being an overproducer of extracellular long-chain AHLs, was superior in rhizoplane colonization. Notably, the *sinI* mutant unable to produce long-chain AHLs (Fig. 8A) showed an impaired rhizoplane colonization rate in the symmetrical inoculation experiment (Fig. S7), whereas abundant *sinI* mutants were identified on the rhizoplane of test plants in the Tn-seq screen (Fig. S10). The Tn-seq result was consistent with that the *sinI* mutant was as competitive as SF2 in terms of rhizoplane colonization rate (*sinI* vs SF2 = 52% vs 48%; *p* > 0.05) when they were inoculated as a 1:1 mixture. In line with these rhizoplane colonization experiments, the *sinI* or *traI sinI* mutants showed impaired surface motility compared to SF2 and the *traI* mutant (Fig. S10B; *p* values < 0.001), but this surface motility defect can be recovered when they were mixed with SF2 (Fig. S10C). Therefore, the rhizoplane colonization defect of the *sinI* mutant was not as significant as that of the mutant lacking a multifunctional ExoF in an inoculant mixture containing the wild-type SF2.

## Conclusion

Engineering root microbiome for sustainable agriculture is still in its infancy, largely due to limited understanding of mechanisms underlying root microbiome assembly [20]. There is an increasing attention on rhizoplane colonization mechanisms of keystone species of root microbiota. Recent pioneer genome-wide Tn-seq analyses are revealing more rhizoplane fitness genes [13–18], functions of which in the migration process from rhizosphere to rhizoplane have not been well addressed. This work focused on a keystone beneficial species *S. fredii*, and performed a genome-wide Tn-seq survey of its genes either positively or negatively modulating rhizoplane colonization on wild soybean, cultivated soybean, rice, and maize (Fig. 1). A robust list of broad-host-range colonization genes was identified (Fig. S2). We then focused on outstanding *fadL* and *exoF*/*exoQ*/*exoP* with negative and positive roles, respectively, in broad-host-range rhizoplane colonization (Fig. 2 and Fig. 3). Reverse genetics characterizations of these four genes and related *exo* genes involved in EPS biosynthesis and degradation suggest that the contrasting rhizoplane colonization ability is mediated by surface motility from rhizosphere to rhizoplane, rather than swimming, and rhizosphere and rhizoplane survivability (Fig. 3 – Fig. 6, and Figs. S4 – S6). With further physiological analyses of quorum-sensing AHLs from these strains (Figs. 7–8), we revealed that FadL mediates the uptake of long-chain AHLs while ExoF likely mediates the secretion of short-chain AHLs, and that accumulation of short-chain AHLs within cells of the *exoF* mutant can lead to down-regulation of biosynthesis and a lower extracellular level of long-chain AHLs (Fig. 7D). Therefore, the contrasting extracellular levels of long-chain AHLs among the *fadL* and *exoF* mutants, and SF2 are in line with the variation in their rhizoplane colonization rates. The critical role of long-chain AHLs in the migration process from rhizosphere to rhizoplane was further verified by the diffusible effect of a synthetic mixture of long-chain AHLs mimicking that of the *fadL* mutant (Fig. 8E, F). Given the wide distribution of FadL and ExoFQP homologs in *Alphaproteobacteria* (Fig. S11), the FadL-ExoFQP mediated regulation of surface motility in the migration toward roots can be an evolutionarily conserved gene-niche interaction mechanism. It shapes the “home life” of rhizobacteria belonging to this class which includes diverse keystone species of root microbiome. The procedure developed in this work can also be used in studies of other bacteria-host interactions. This work and other published high-throughput studies on rhizoplane fitness genes are still restricted to the well-known keystone species under well-controlled laboratory conditions. Although ecological roles of these keystone species in soil-plant-microbiota interactions have been intensively studied, functions of rhizoplane fitness genes have been rarely further tested under complicated and fluctuating soil conditions which intensively and dynamically interact with plants and microbiota. In contrast to the fast cumulation of metagenomic data related to root microbiome research, our functional characterization efforts are still limited, and high-throughput functional genomics tools (e.g., Tn-seq) should be further improved and explored under various soil conditions.

## Supplementary information

Supplementary Figure S1

Supplementary Figure S2

Supplementary Figure S3

Supplementary Figure S4

Supplementary Figure S5

Supplementary Figure S6

Supplementary Figure S7

Supplementary Figure S8

Supplementary Figure S9

Supplementary Figure S10

Supplementary Figure S11

Supplementary Table S1

Supplementary Table S2

Supplementary Table S3

Supplementary Table S4

Supplementary Data S1

Supplementary Data S2

## Data Availability

Raw sequence data from our Tn-seq analysis can be accessed via NCBI Sequence Read Archive (PRJNA808870).
