# Supplementary material for: Rhizobial migration toward roots mediated by FadL-ExoFQP modulation of extracellular long-chain AHLs
Source: ISME J. 2023 Jan 10;17(3):417–31. doi: 10.1038/s41396-023-01357-5 (PMC9938287; doi:10.1038/s41396-023-01357-5)
Supplement: Supplementary file 1 — Supplementary Figure S1 [file 41396_2023_1357_MOESM1_ESM.pdf]

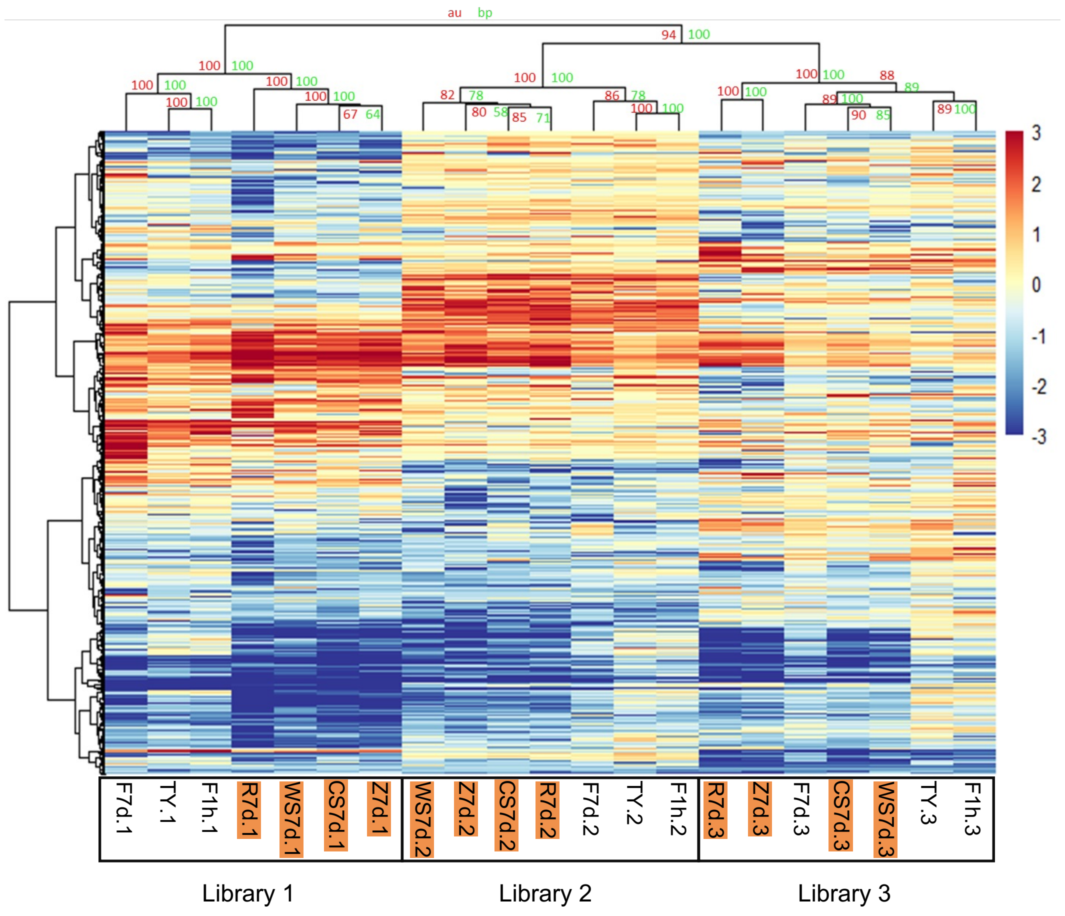

Fig. S1. Stochastic effect of three independent input libraries. Heatmap of gene fitness scores higher than 2.4 or lower than -2.4 (average fitness score ± 3σ) under at least one condition compared to the corresponding input library. Approximately unbiased probability and bootstrap probability (au/bp) values are shown. Rhizoplane samples are indicated by orange background.
