# Supplementary material for: Rhizobial migration toward roots mediated by FadL-ExoFQP modulation of extracellular long-chain AHLs
Source: ISME J. 2023 Jan 10;17(3):417–31. doi: 10.1038/s41396-023-01357-5 (PMC9938287; doi:10.1038/s41396-023-01357-5)
Supplement: Supplementary file 2 — Supplementary Figure S2 [file 41396_2023_1357_MOESM2_ESM.pdf]

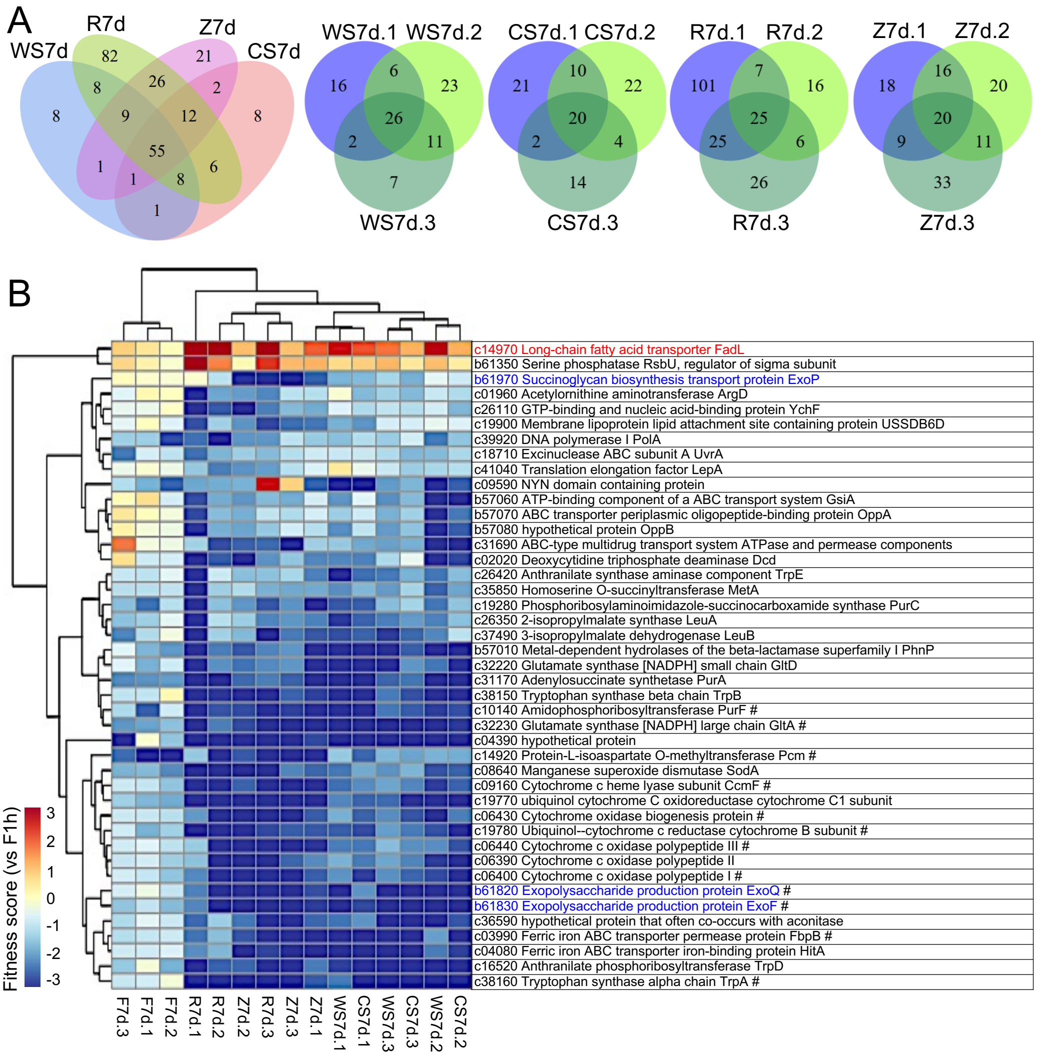

Fig. S2. Mutants with reduced or enhanced fitness scores in output samples of three independent experiments. The fitness scores of F7d, WS7d, CS7d, R7d, and Z7d compared to F1h. (**A**) Venn diagram showing the number of genes involved in rhizoplane colonization on different plant roots in three independent experiments. (**B**) Heatmap of fitness scores for 43 genes recurrently identified as a fitness player in three independent experiments (significant for at least one test plant species; *p* values < 0.01). #, statistically significant in all rhizoplane samples.
