# Supplementary material for: Rhizobial migration toward roots mediated by FadL-ExoFQP modulation of extracellular long-chain AHLs
Source: ISME J. 2023 Jan 10;17(3):417–31. doi: 10.1038/s41396-023-01357-5 (PMC9938287; doi:10.1038/s41396-023-01357-5)
Supplement: Supplementary file 3 — Supplementary Figure S3 [file 41396_2023_1357_MOESM3_ESM.pdf]

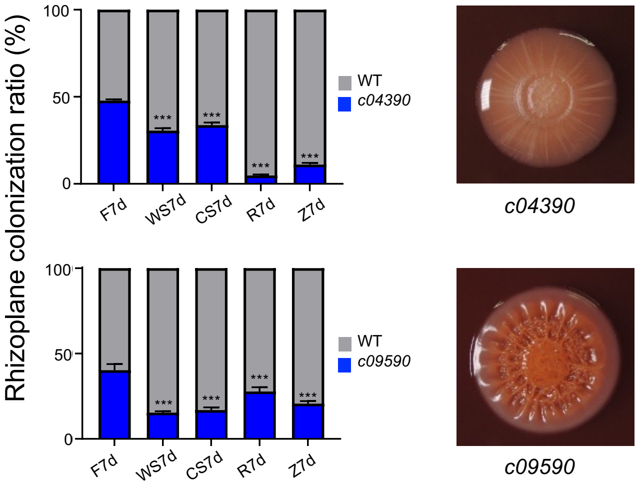

Fig. S3. Impaired rhizoplane colonization ability of the *c04390* and *c09590* mutants. The mutant was co-inoculated with the wild-type strain SF2 at 1:1 ratio under the same conditions used for Tn-seq (Fig 1). Significant difference is indicated (one sample *t* test; theoretical mean = 0.5; ***, $p < 0.001$). Error bars represent SD of three biological replicates. Colonies of two mutants on the surface motility plate (the TY medium containing 0.5% agar and Congo Red) are shown.
