# Supplementary material for: Rhizobial migration toward roots mediated by FadL-ExoFQP modulation of extracellular long-chain AHLs
Source: ISME J. 2023 Jan 10;17(3):417–31. doi: 10.1038/s41396-023-01357-5 (PMC9938287; doi:10.1038/s41396-023-01357-5)
Supplement: Supplementary file 4 — Supplementary Figure S4 [file 41396_2023_1357_MOESM4_ESM.pdf]

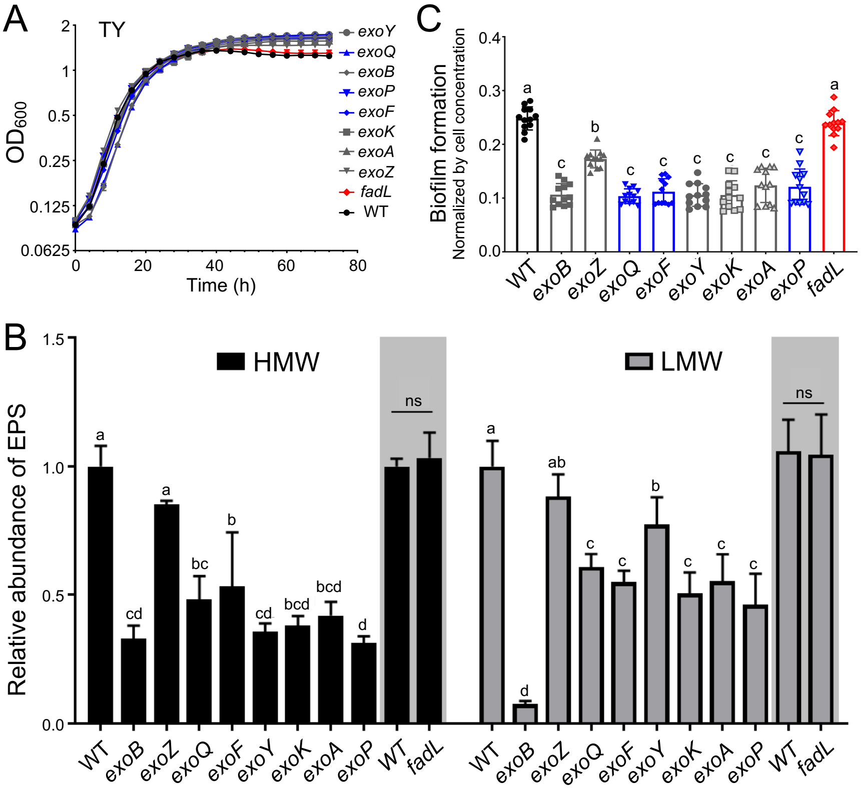

Fig. S4. Variations in exopolysaccharide production and biofilm formation. (**A**) Growth curves in the TY rich medium (mean ± SEM of three biological replicates). (**B**) Production of high (HMW) and low molecular weight (LMW) extracellular polysaccharides (EPS). Gray shaded area indicates the results of independent experiments for WT and the *fadL* mutant. (**C**) Biofilm formation. (**B**-**C**) Different letters indicate significant difference between means (± SEM; ANOVA followed by Duncan's test, alpha = 0.05; three independent experiments).
