# Supplementary material for: Rhizobial migration toward roots mediated by FadL-ExoFQP modulation of extracellular long-chain AHLs
Source: ISME J. 2023 Jan 10;17(3):417–31. doi: 10.1038/s41396-023-01357-5 (PMC9938287; doi:10.1038/s41396-023-01357-5)
Supplement: Supplementary file 5 — Supplementary Figure S5 [file 41396_2023_1357_MOESM5_ESM.pdf]

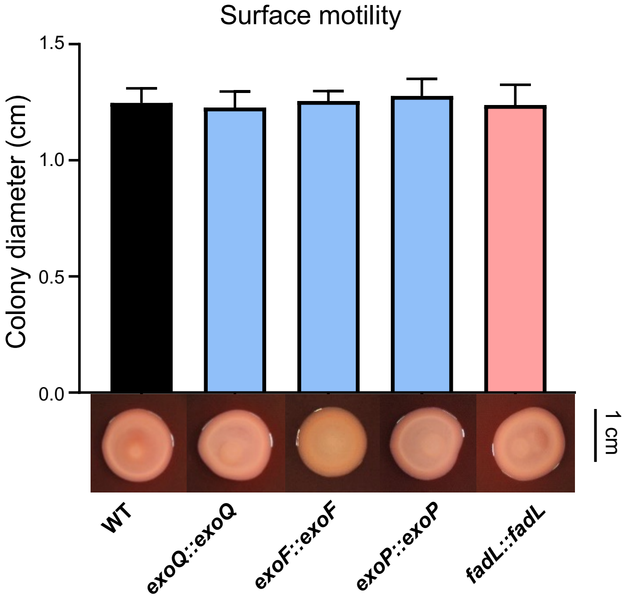

Fig. S5. Surface motility of complementary mutant strains. The TY medium (0.5% agar with Congo red) was used. No significant difference was observed between the wild-type SF2 (WT) and complementary mutant strains (ANOVA followed by Duncan's test, alpha = 0.05). Error bars represent SEM of three biological replicates.
