# Supplementary material for: Rhizobial migration toward roots mediated by FadL-ExoFQP modulation of extracellular long-chain AHLs
Source: ISME J. 2023 Jan 10;17(3):417–31. doi: 10.1038/s41396-023-01357-5 (PMC9938287; doi:10.1038/s41396-023-01357-5)
Supplement: Supplementary file 6 — Supplementary Figure S6 [file 41396_2023_1357_MOESM6_ESM.pdf]

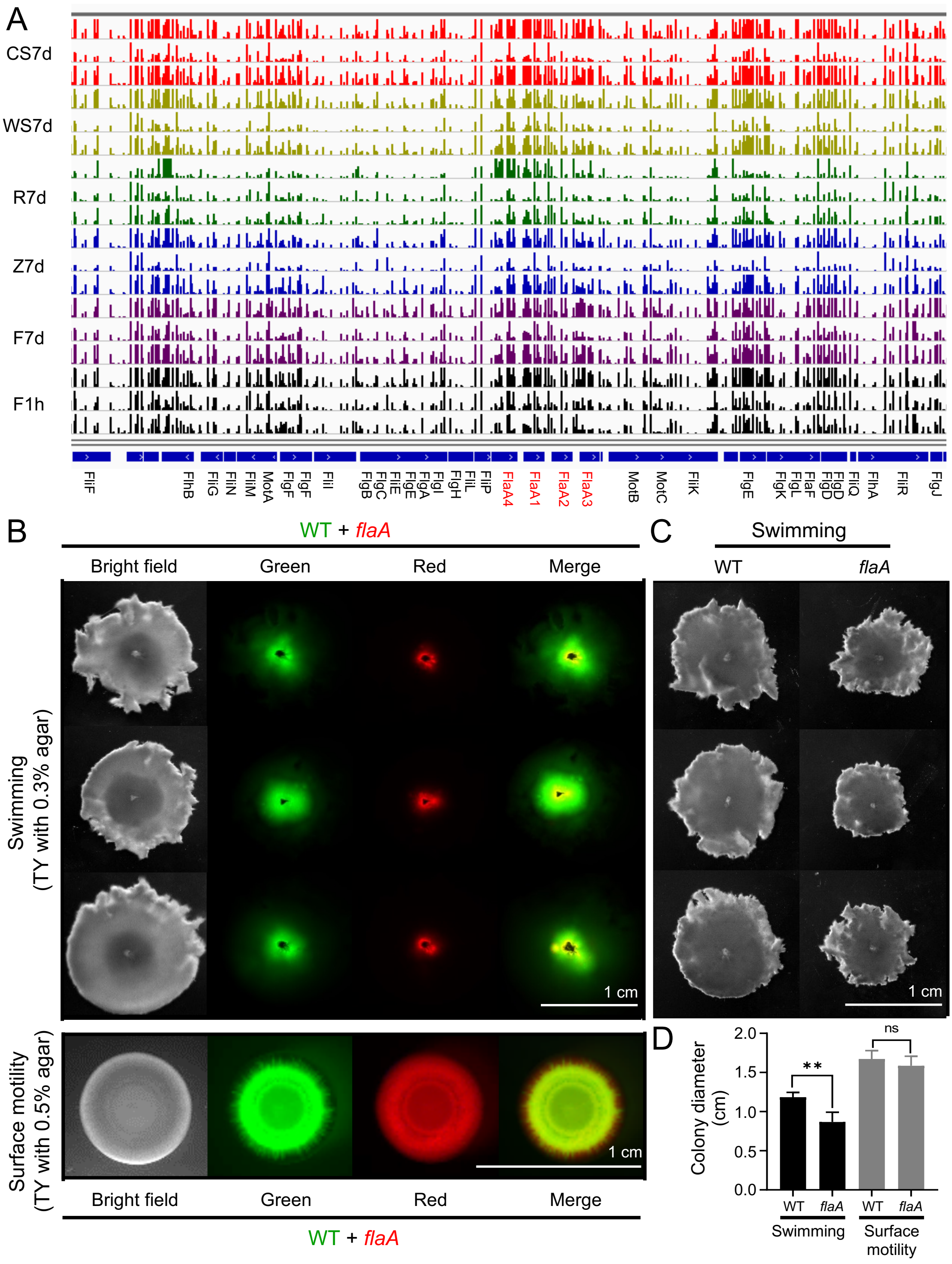

Fig. S6. Tn-seq reads mapped in the flagellar biosynthesis gene cluster and motility phenotypes of the *flaA* mutant. (**A**) The transposon insertion frequency in the flagellar biosynthesis gene cluster under different conditions in three independent experiments (rows). (**B**) Fluorescence stereo microscopy pictures of swimming and surface motility of the *flaA* mutant (red) compared to the wild-type SF2 (WT; green) in an 1:1 mixture. The *flaA* mutant lacks four flagellin genes (*flaA4, flaA1, flaA2,* and *flaA3*). (**C**) The swimming phenotypes of the *flaA* mutant and WT. (**D**) Statistical analysis of swimming ability (TY with 0.3% agar) and surface motility (TY plate containing 0.5% agar and Congo red) of the *flaA* mutant compared to WT. Significant difference between means are indicated (**, $p < 0.01$; ns, not significant; *t* test), and error bars represent SEM of three biological replicates.
