# Supplementary material for: Rhizobial migration toward roots mediated by FadL-ExoFQP modulation of extracellular long-chain AHLs
Source: ISME J. 2023 Jan 10;17(3):417–31. doi: 10.1038/s41396-023-01357-5 (PMC9938287; doi:10.1038/s41396-023-01357-5)
Supplement: Supplementary file 7 — Supplementary Figure S7 [file 41396_2023_1357_MOESM7_ESM.pdf]

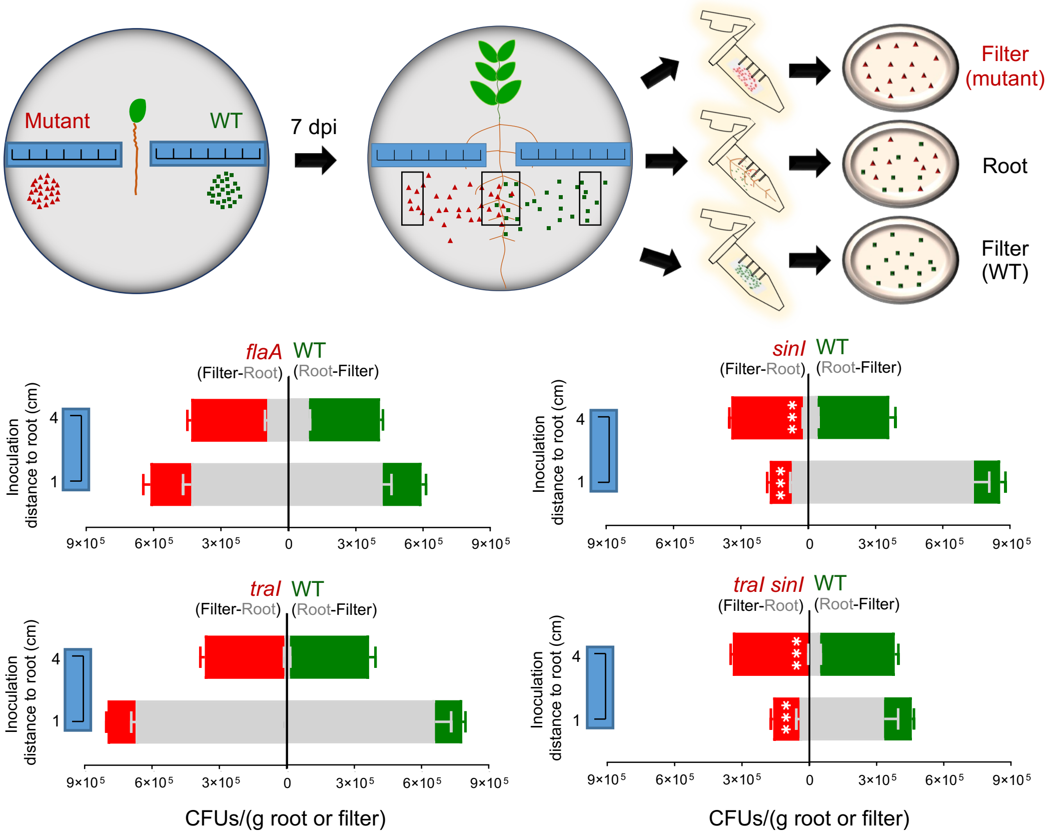


**Fig. S7. The rhizoplane colonization ability of *flaA*, *traI*, *sinI*, and *traI sinI* mutants.** Colony forming units (CFUs) from the inoculation sites (filter; 1 cm and 4 cm away from the root) and rhizoplane (root) were tested at 7 dpi (days post inoculation). Significant difference between means of the mutant and WT are indicated (***, $p < 0.001$; $t$ test), and error bars represent SEM of three biological replicates.
