# Supplementary material for: Rhizobial migration toward roots mediated by FadL-ExoFQP modulation of extracellular long-chain AHLs
Source: ISME J. 2023 Jan 10;17(3):417–31. doi: 10.1038/s41396-023-01357-5 (PMC9938287; doi:10.1038/s41396-023-01357-5)
Supplement: Supplementary file 8 — Supplementary Figure S8 [file 41396_2023_1357_MOESM8_ESM.pdf]

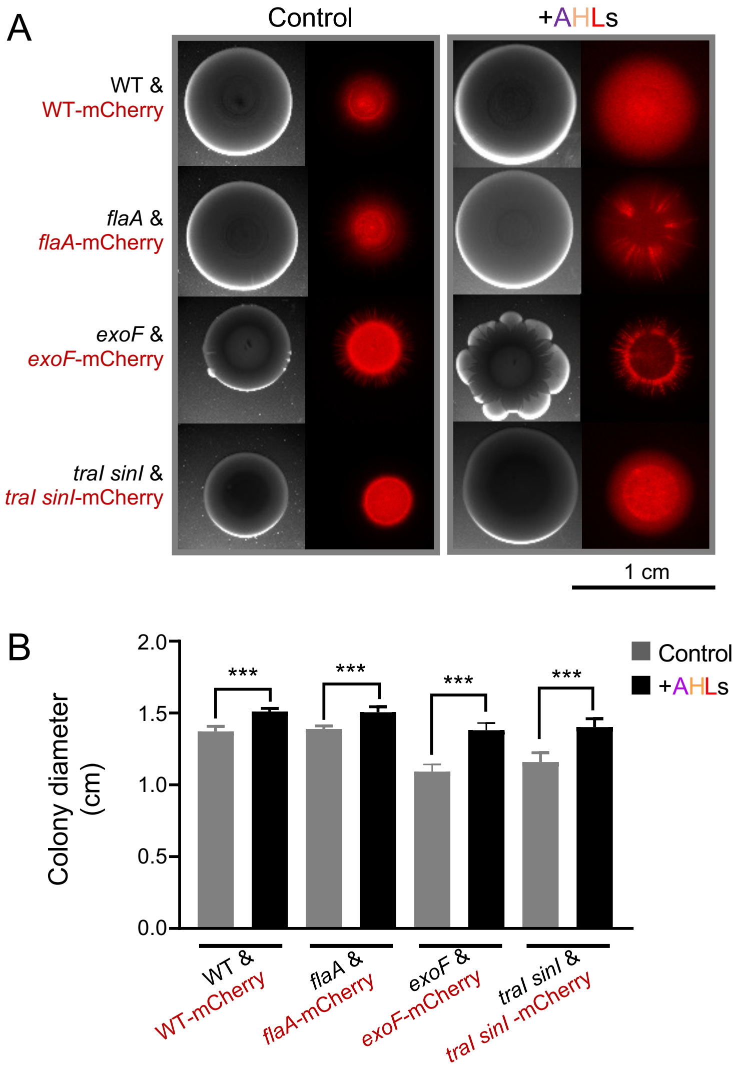

Fig. S8. Long-chain AHLs enhance surface motility of rhizobia. (**A**) Fluorescence stereo microscopy pictures of surface motility ability of the mCherry labeled strain and unlabeled strain in an 1:1 mixture on the TY plate (0.5% agar with Congo red). The bacterial mixture was supplemented with or without 2 µl long-chain AHLs (45 ng 3-OXO-C12-HSL, 52 ng C14-HSL, and 1 µg 3-OXO-C14-HSL). (**B**) Statistical analysis of surface motility based on diameter of colonies from (**A**). Significant difference between means are indicated (***, p < 0.001; t test), and error bars represent SEM of three biological replicates.
