# Supplementary material for: Rhizobial migration toward roots mediated by FadL-ExoFQP modulation of extracellular long-chain AHLs
Source: ISME J. 2023 Jan 10;17(3):417–31. doi: 10.1038/s41396-023-01357-5 (PMC9938287; doi:10.1038/s41396-023-01357-5)
Supplement: Supplementary file 9 — Supplementary Figure S9 [file 41396_2023_1357_MOESM9_ESM.pdf]

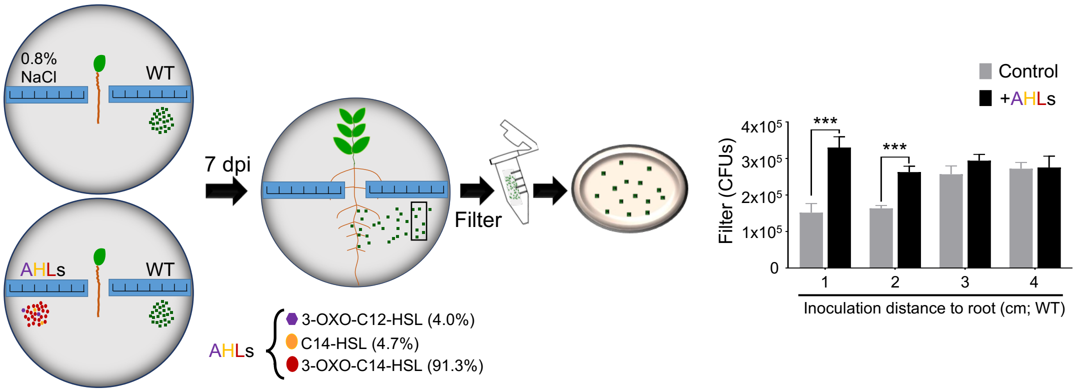

Fig. S9. Long-chain AHLs promote rhizosphere survival of the wild-type SF2 (*G. soja* plants). Long-chain AHLs were mixed according to extracellular AHLs composition detected from the *fadL* mutant (2μl long-chain AHLs solution containing 45 ng 3-OXO-C12-HSL, 52 ng C14-HSL, and 1 μg 3-OXO-C14-HSL). Significant difference between means are indicated (***, $p < 0.001$; t test; ±SEM based on three independent experiments).
