# Supplementary material for: Rhizobial migration toward roots mediated by FadL-ExoFQP modulation of extracellular long-chain AHLs
Source: ISME J. 2023 Jan 10;17(3):417–31. doi: 10.1038/s41396-023-01357-5 (PMC9938287; doi:10.1038/s41396-023-01357-5)
Supplement: Supplementary file 10 — Supplementary Figure S10 [file 41396_2023_1357_MOESM10_ESM.pdf]

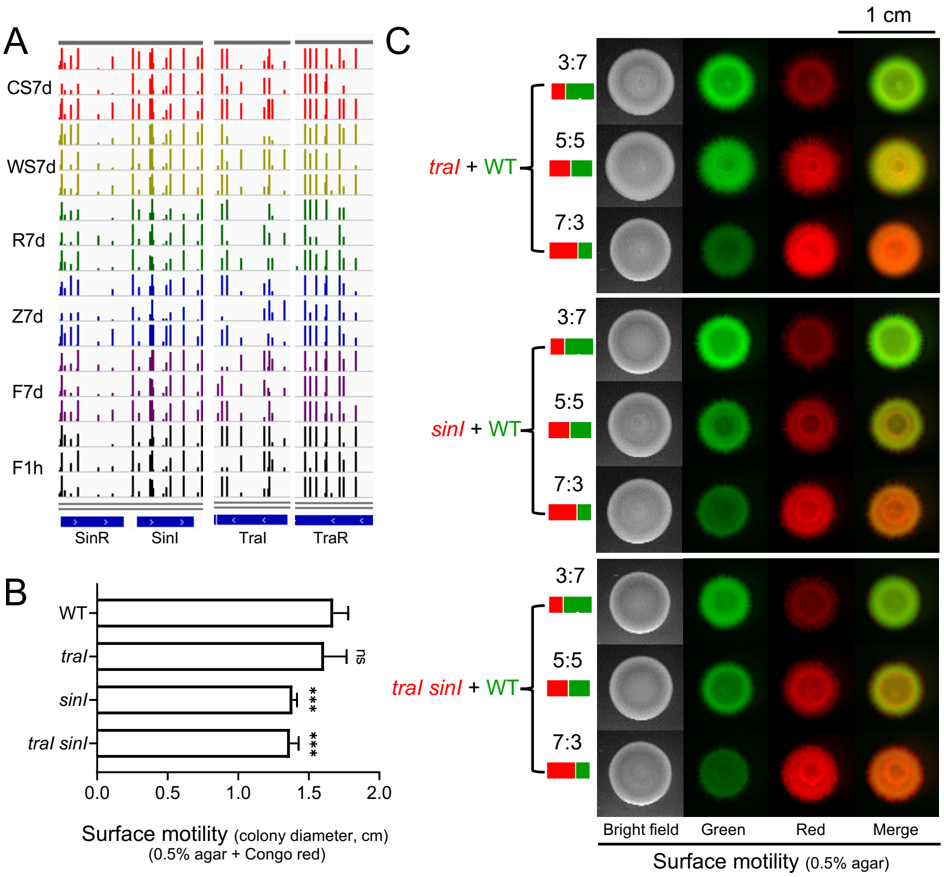


**Fig. S10. Tn-seq reads mapped in *sinR*, *sinI*, *traR*, and *traI,* and motility phenotypes of related mutants.** (**A**) The transposon insertion frequency in genes related to the synthesis (*sinI* and *traI*) and regulation (*sinR* and *traR*) of long-chain (*sinI* and *sinR*) and short-chain (*traI* and *traR*) AHLs under different conditions in three independent experiments (rows; reads mapped in the same gene from different samples are at the same scale). (**B**) Surface motility determined by diameter of colonies on the TY plate (0.5% agar with Congo red). Significant difference between means of the mutant and WT are indicated (***, $p < 0.001$; ns, not significant; *t* test), and error bars represent SEM of three biological replicates. (**C**) Fluorescence stereo microscopy pictures of surface motility of the mutant (red) compared to SF2 (WT; green) in an inoculant mixture (mixed in different ratios as follows: 3:7, 5:5, and 7:3) on the TY plate (0.5% agar).
