# Supplementary material for: Rhizobial migration toward roots mediated by FadL-ExoFQP modulation of extracellular long-chain AHLs
Source: ISME J. 2023 Jan 10;17(3):417–31. doi: 10.1038/s41396-023-01357-5 (PMC9938287; doi:10.1038/s41396-023-01357-5)
Supplement: Supplementary file 11 — Supplementary Figure S11 [file 41396_2023_1357_MOESM11_ESM.pdf]

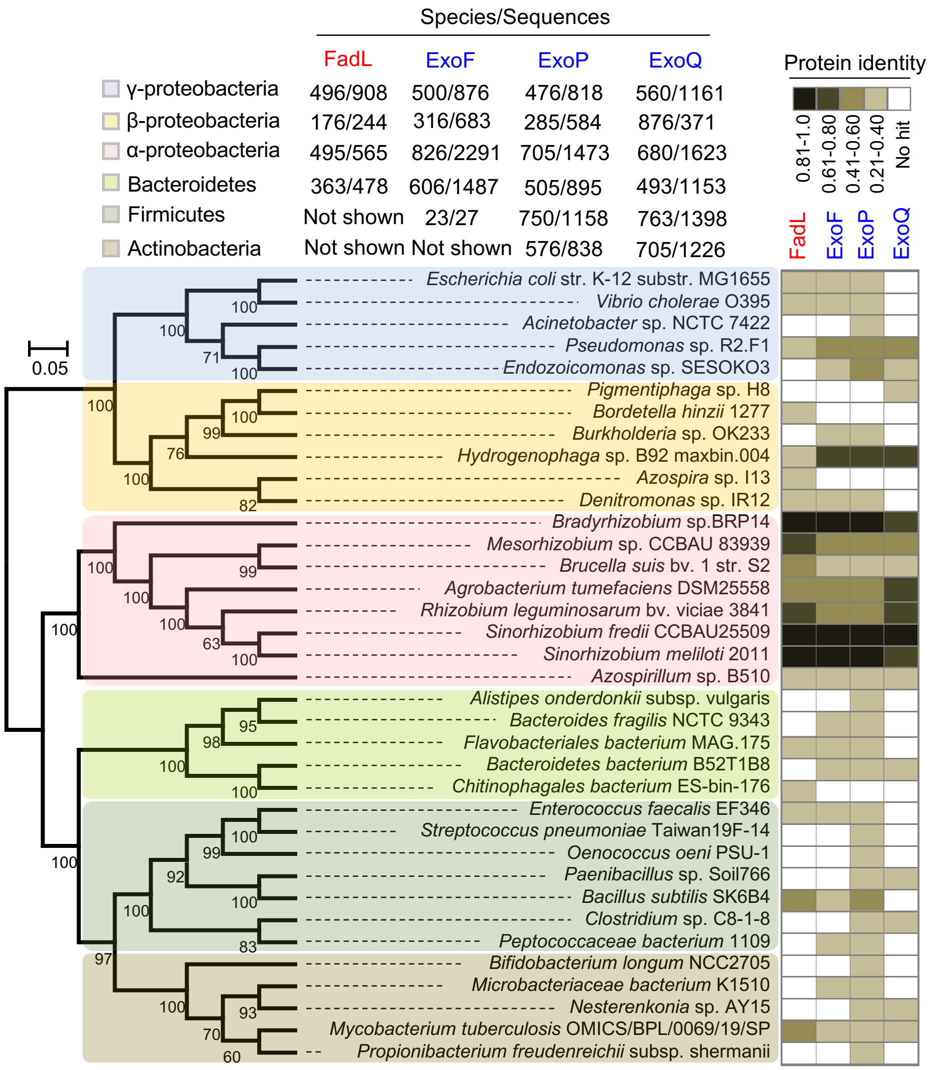

Fig. S11. Phyletic distribution of homologs of FadL, ExoF, ExoP, and ExoQ. The neighbor-joining phylogenetic tree was constructed based on RpoB. The number of species containing corresponding homologs and the number of protein sequences available in the Pfam database are shown. The protein identity values of homologs are indicated.
