# Supplementary material for: Rhizobial migration toward roots mediated by FadL-ExoFQP modulation of extracellular long-chain AHLs
Source: ISME J. 2023 Jan 10;17(3):417–31. doi: 10.1038/s41396-023-01357-5 (PMC9938287; doi:10.1038/s41396-023-01357-5)
Supplement: Supplementary file 14 — Supplementary Table S3 [file 41396_2023_1357_MOESM14_ESM.docx]

**Table S3 Summary statistics of Tn-seq analysis.**

| **Treatment** | **Library** | **Reads containing Tn end** | **Reads mapping to genome** | **No. of insertions** | **Unique insertions** | **Insertions per TA** | **Insertion density** | **Genes disrupted** |
| --- | --- | --- | --- | --- | --- | --- | --- | --- |
| **INPUT** | **Lib_1** | **12852517** | **11667208** | **8660912** | **93942** | **92** | **82.73%** | **6621** |
|  | **Lib_2** | **2572407** | **2312935** | **1736967** | **64754** | **27** | **57.03%** | **6348** |
|  | **Lib_3** | **34901987** | **31538613** | **23208388** | **99469** | **233** | **87.60%** | **6623** |
| **TY** | **Lib_1** | **24575005** | **22502973** | **16638993** | **94153** | **177** | **82.92%** | **6563** |
|  | **Lib_2** | **12291952** | **11562990** | **8835818** | **84517** | **105** | **74.43%** | **6471** |
|  | **Lib_3** | **29436463** | **15263352** | **11542516** | **93326** | **124** | **82.19%** | **6533** |
| **F1h** | **Lib_1** | **18202560** | **14406066** | **10916539** | **88702** | **123** | **78.12%** | **6524** |
|  | **Lib_2** | **16156584** | **15461439** | **11958459** | **86682** | **138** | **76.34%** | **6499** |
|  | **Lib_3** | **15773178** | **14937573** | **11376052** | **91582** | **124** | **80.65%** | **6514** |
| **F7d** | **Lib_1** | **43494020** | **39776599** | **29530466** | **98781** | **299** | **86.99%** | **6660** |
|  | **Lib_2** | **15310642** | **13888760** | **10678434** | **83808** | **127** | **73.81%** | **6469** |
|  | **Lib_3** | **17038107** | **16329439** | **12369636** | **93225** | **133** | **82.10%** | **6540** |
| **R7d** | **Lib_1** | **18908349** | **18069603** | **13683623** | **78377** | **175** | **69.03%** | **6388** |
|  | **Lib_2** | **17372100** | **16523759** | **12944469** | **76118** | **170** | **67.04%** | **6388** |
|  | **Lib_3** | **14749325** | **14189581** | **11015492** | **81265** | **136** | **71.57%** | **6407** |
| **CS7d** | **Lib_1** | **21774131** | **20137628** | **14999612** | **89187** | **168** | **78.55%** | **6478** |
|  | **Lib_2** | **16457431** | **15202276** | **11943848** | **77092** | **155** | **67.89%** | **6393** |
|  | **Lib_3** | **24957717** | **23751711** | **18072655** | **93528** | **193** | **82.37%** | **6536** |
| **WS7d** | **Lib_1** | **18466621** | **17789059** | **13666813** | **89068** | **153** | **78.44%** | **6496** |
|  | **Lib_2** | **12970249** | **12450910** | **9675615** | **77242** | **125** | **68.03%** | **6399** |
|  | **Lib_3** | **12213975** | **11908579** | **9095486** | **89523** | **102** | **78.84%** | **6465** |
| **Z7d** | **Lib_1** | **16874007** | **16009422** | **12193092** | **85070** | **143** | **74.92%** | **6483** |
|  | **Lib_2** | **20014762** | **14465895** | **11304342** | **70850** | **160** | **62.40%** | **6331** |
|  | **Lib_3** | **25086255** | **24161698** | **18842753** | **86564** | **218** | **76.24%** | **6490** |
