# Supplementary material for: Rhizobial migration toward roots mediated by FadL-ExoFQP modulation of extracellular long-chain AHLs
Source: ISME J. 2023 Jan 10;17(3):417–31. doi: 10.1038/s41396-023-01357-5 (PMC9938287; doi:10.1038/s41396-023-01357-5)
Supplement: Supplementary file 15 — Supplementary Table S4 [file 41396_2023_1357_MOESM15_ESM.docx]

**Table S4 Symbiotic performance of *Sinorhizobium fredii* CCBAU25509 and its mutants on wild soybean plants.**

| **Treatment** | **Chlorophyll content (SPAD value)** | **Shoot dry weight (g/plant)** | **Nodule number (per plant)** |
| --- | --- | --- | --- |
|  |  |  |  |
| **WT** | 44.42±1.89 (a) | 0.63±0.07 (a) | 15.07±8.38 (a) |
| ***exoB*** | 22.98±3.54 (b) | 0.36±0.09 (b) | 3.14±1.61 (b) |
| ***exoZ*** | 42.96±1.32 (a) | 0.61±0.11 (a) | 14.07±2.27 (a) |
| ***exoQ*** | 43.28±2.26 (a) | 0.62±0.12 (a) | 15.93±5.09 (a) |
| ***exoF*** | 43.37±1.56 (a) | 0.57±0.09 (a) | 12.29±3.20 (a) |
| ***exoY*** | 43.28±1.22 (a) | 0.61±0.10 (a) | 15.71±4.46 (a) |
| ***exoK*** | 43.77±1.10 (a) | 0.57±0.09 (a) | 12.93±4.27 (a) |
| ***exoA*** | 43.80±1.63 (a) | 0.59±0.06 (a) | 12.71±3.50 (a) |
| ***exoP*** | 42.97±1.81 (a) | 0.57±0.09 (a) | 13.86±4.55 (a) |
| ***fadL*** | 42.88±1.94 (a) | 0.61±0.08 (a) | 14.43±3.69 (a) |
| **Control** | 10.49±1.36 (c) | 0.30±0.06 (b) |  |

Different letters in brackets indicate significant difference between treatments (Average ± SD; ANOVA followed by Duncan's test, alpha = 0.05). More than 15 plants were scored.
